# Mu opioid receptors on vGluT2‐expressing glutamatergic neurons modulate opioid reward

**DOI:** 10.1111/adb.12942

**Published:** 2020-07-20

**Authors:** Kaitlin C. Reeves, Megan J. Kube, Gregory G. Grecco, Brandon M. Fritz, Braulio Muñoz, Fuqin Yin, Yong Gao, David L. Haggerty, Hunter J. Hoffman, Brady K. Atwood

**Affiliations:** ^1^ Department of Pharmacology and Toxicology Indiana University School of Medicine Indianapolis Indiana USA; ^2^ Medical Scientist Training Program Indiana University School of Medicine Indianapolis Indiana USA; ^3^ Stark Neurosciences Research Institute Indiana University School of Medicine Indianapolis Indiana USA

**Keywords:** electrophysiology, glutamate, Mu opioid receptor, reward, transgenic mice, vGluT2

## Abstract

The role of Mu opioid receptor (MOR)‐mediated regulation of GABA transmission in opioid reward is well established. Much less is known about MOR‐mediated regulation of glutamate transmission in the brain and how this relates to drug reward. We previously found that MORs inhibit glutamate transmission at synapses that express the Type 2 vesicular glutamate transporter (vGluT2). We created a transgenic mouse that lacks MORs in vGluT2‐expressing neurons (MORflox‐vGluT2cre) to demonstrate that MORs on the vGluT2 neurons themselves mediate this synaptic inhibition. We then explored the role of MORs in vGluT2‐expressing neurons in opioid‐related behaviors. In tests of conditioned place preference, MORflox‐vGluT2cre mice did not acquire place preference for a low dose of the opioid, oxycodone, but displayed conditioned place aversion at a higher dose, whereas control mice displayed preference for both doses. In an oral consumption assessment, these mice consumed less oxycodone and had reduced preference for oxycodone compared with controls. MORflox‐vGluT2cre mice also failed to show oxycodone‐induced locomotor stimulation. These mice displayed baseline withdrawal‐like responses following the development of oxycodone dependence that were not seen in littermate controls. In addition, withdrawal‐like responses in these mice did not increase following treatment with the opioid antagonist, naloxone. However, other MOR‐mediated behaviors were unaffected, including oxycodone‐induced analgesia. These data reveal that MOR‐mediated regulation of glutamate transmission is a critical component of opioid reward.

## Graphical abstract

In this study, the authors created a transgenic mouse line that lacked expression of Mu opioid receptors only in a subtype of glutamate neurons that express the Type 2 vesicular glutamate transporter. These mice had disrupted behaviors related to opioid reward but not ethanol or natural reward and had intact opioid analgesic responses. These findings indicate that Mu opioid receptors in glutamate neurons are major contributors to opioid reward related behaviors.

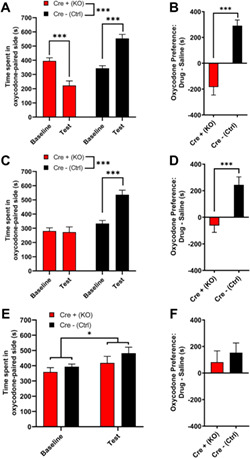

## 1 INTRODUCTION

The abuse of opioids, such as oxycodone, is on the rise in the United States, with more than 47,000 deaths due to opioid overdose in 2017.^1^ Approximately one quarter of people prescribed opioids for pain management abuse them.^2^ Chronic opioid abuse results in neurobehavioral changes including increased risk taking, impaired working memory, and impaired cognitive performance.^3^ Mu opioid receptors (MORs) mediate the rewarding and analgesic effects of commonly prescribed and abused opioids.^4^ It is well established that MORs modulate opioid reward through inhibition of GABA transmission.^5^ The classical model of opioid reward implicates MOR‐mediated depression of GABA release from VTA GABAergic interneurons that synapse onto local ventral tegmental area (VTA) dopamine neurons. MOR activation decreases the inhibitory tone to VTA dopamine neurons, resulting in increased dopamine release in the nucleus accumbens (NAc).^5^ This model is supported by the fact that ablating MOR expression in the VTA blocks the rewarding and stimulating effects of opioids. Others have suggested a revised model where MORs on GABAergic inputs from other brain regions to VTA dopamine neurons may mediate behavioral responses to opioids. In addition, other studies indicate that MORs expressed in forebrain GABA neurons also modulate opioid reward.^6^, ^7^

MORs also regulate glutamate transmission in multiple neurocircuits, including those involved in drug abuse, but their specific role in opioid reward is unknown.^8^, ^9^, ^10^, ^11^, ^12^, ^13^, ^14^, ^15^ It is increasingly clear that glutamate transmission is a critical component of opioid reward‐related behaviors. The illicit opioid, heroin, increases extracellular glutamate in the NAc, an effect not seen with feeding‐related behaviors.^16^, ^17^ Within the NAc and VTA, acute opioid exposure depresses glutamate release but enhances NMDA glutamate receptor function. Morphine fails to promote dopamine neuron activation following inhibition of VTA AMPA and NMDA receptors.^18^, ^19^ This treatment also inhibits the development of morphine conditioned place preference (CPP) without affecting locomotor activity.^20^

We previously showed that MOR activation inhibits multiple glutamatergic inputs to the striatum, a critical brain region controlling behavioral responses to drugs of abuse. In these studies, we found that MOR activation inhibits glutamate transmission at Type 2 vesicular glutamate transporter (vGluT2)‐expressing thalamic inputs to striatal medium spiny neurons.^21^ vGluT2‐expressing neurons are a subclass of glutamate neurons widely expressed throughout the brain.^22^ vGluT2‐expressing neurons in multiple brain regions modulate reward‐related behaviors.^23^, ^24^, ^25^, ^26^ Given our findings that MORs regulate glutamate transmission at vGluT2‐expressing synapses and the involvement of vGluT2 neurons in reward‐related neurocircuitry, we decided to investigate the role of MORs in vGluT2 neurons in behavioral responses to opioids. Here, we report on the generation of a mutant mouse that selectively lacks MOR expression within vGluT2 neurons and its behavioral responses to the prescription opioid analgesic, oxycodone.

## 2 MATERIALS AND METHODS

### 2.1 Animals

Adult male and female C57BL/6J mice were purchased from Jackson Labs (JAX #000664). MORflox‐vGluT2cre mice were bred and genotyped in‐house from conditional MOR knockout mice (MORflox) and vGluT2cre progenitors (vGlut2Cre: JAX #016963). MORflox mice were generously donated by Dr Jennifer Whistler (UC Davis) and were previously characterized.^21^, ^27^ All experimental protocols used in this study were approved by the Institutional Animal Care and Use Committee at the Indiana University School of Medicine, and all guidelines for ethical treatment and care for experimental animals established by the National Institutes of Health (NIH, Maryland, USA) were followed. Additional details may be found in the supporting information.

### 2.2 Materials

Oxycodone hydrochloride (Sigma Aldrich, St. Louis, MO) was dissolved in saline (0.9% w/v) for in vivo injections and in reverse‐osmosis water for drinking studies. DAMGO was purchased from Bachem (Torrance, CA). All other chemicals and reagents were purchased from Sigma‐Aldrich (St. Louis, MO) or ThermoFisher (Waltham, MA).

### 2.3 Measurement of MOR expression

#### 2.3.1 Tissue dissection, RNA extraction, and reverse transcription

Adult male mice were deeply anesthetized via isoflurane and whole brains were rapidly excised. Following the Franklin and Paxinos atlas (*The Mouse Brain*, Elsevier, 2007), sections containing thalamus or prefrontal cortex (including medial prefrontal, orbitofrontal, and anterior insular cortices) were made using a stainless steel mouse brain matrix (Zivic Instruments, Pittsburgh, PA) and were rapidly dissected and collected from each mouse. The dissected tissues were immediately homogenized, and RNA was isolated using the RNeasy Plus Universal Mini kit (Qiagen; Cat No: 73404) according to the manufacturer's protocol. RNA concentrations were determined by Nanodrop (ThermoFisher), and 1 μg of total RNA was converted to complementary DNA (cDNA) using the High Capacity cDNA Reverse Transcription kit (ThermoFisher; Cat No: 4368814).

#### 2.3.2 mRNA quantification

We used quantitative polymerase chain reaction (qPCR) to determine MOR gene expression. The qPCR primers used for the MOR gene (mus musculus opioid receptor, μ1 [Oprm1], transcript variant MOR‐1C, mRNA) were developed and ordered from Integrated DNA Technologies (Coralville, IA, USA). The forward primer (5′‐CTCCTGGCTCAACTTGTCCCACGT ‐3′) targeted a portion of exon 1, and the reverse primer (5′‐ ACAGTGATGATGAGGACCGGCATG‐3′) targeted a portion of exon 3. The specificity of the primers was verified through PCR using thalamus cDNA, and the PCR product was subcloned via the TOPO TA cloning kit (ThermoFisher, Cat No: 450071) to confirm the sequence. qPCR was conducted by using the SsoAdvanced Universal SYBR Green Supermix (Bio‐Rad, Hercules, CA; Cat No: 1725270) on a CFX Connect Real‐Time PCR Detection System (Bio‐Rad). The relative amount of each transcript was determined via normalization across all samples to the endogenous control, GAPDH. RNA samples from each individual animal were run in triplicate. To quantify the relative expression levels of the genes for each mouse genotype, we calculated the difference (ΔCt) between the cycle threshold of Oprm1 and the housekeeping gene, GAPDH. From these data, the ΔΔCt ([ΔCtOprm1(Cre+) − ΔCtOprm1(Cre−)]) was computed and converted to a relative quantitative (RQ) value using the formula 2^−ΔΔCt^.

### 2.4 Stereotaxic surgery

Male MORflox‐vGluT2cre mice and littermate controls, at postnatal days 62‑80, were anesthetized with isoflurane and stereotaxically injected with the adeno‐associated viral (AAV) vector, AAV9.hSyn.ChR2(H134R)‐eYFP (Addgene) to drive expression of the photosensitive cation channel, channelrhodopsin2 (ChR2), in thalamic neurons. Bilateral injections were made into the thalamus (coordinates: A/P: −1.6, M/L: ±0.35, D/V: −3.5) (100 nl/injection, 25nl/min infusion rate). Mice were allowed to recover for at least 2 weeks before electrophysiological recordings.

### 2.5 Electrophysiology

Brains slices were prepared for electrophysiological recordings as previously described.^21^ Additional details may be found in the supporting information. Optically evoked excitatory postsynaptic currents (oEPSCs) in dorsal striatal medium spiny neurons were produced in brain slices using 470nm blue light (5 ms) delivered via field illumination through the microscope objective. Light intensity was adjusted to produce stable oEPSCs of 200 to 600pA amplitude prior to experimental recording. oEPSCs were evoked every 30 s. Prior to recording, brain slices were imaged via an Olympus MVX10 microscope (Olympus Corporation of America, Center Valley, PA) to verify ChR2‐eYFP expression. Whole cell recordings of oEPSCs were carried out at 29–32 C°, and aCSF was continuously perfused at a rate of 1 to 2 ml/min. Recordings were performed in the voltage clamp configuration using a Multiclamp 700B amplifier and a Digidata 1550B (Molecular Devices, San Jose, CA). Slices were visualized on an Olympus BX51WI microscope. Medium spiny neurons were identified by their size, membrane resistance, and capacitance. Picrotoxin (50 μM) was added to the aCSF for recordings to isolate excitatory transmission. Patch pipettes were prepared from filament‐containing borosilicate micropipettes (World Precision Instruments, Sarasota, FL) using a P‐1000 micropipette puller (Sutter Instruments, Novato, CA), having a 2.0 to 3.5MΩ resistance. The internal solution contained (in mM): 120 CsMeSO3, 5 NaCl, 10 TEA‐Cl, 10 HEPES, 5 lidocaine bromide, 1.1 EGTA, 0.3 Na‐GTP, and 4 Mg‐ATP (pH 7.2 and 290‑310 mOsm). Neurons were voltage clamped at −60 mV for the duration of the recordings. Data were acquired using Clampex 10.3 (Molecular Devices). Series resistance was monitored, and only cells with a stable series resistance (less than 25 MΩ and that did not change more than 15% during recording) were included for data analysis. Recordings were made 2 to 7 h after euthanasia.

### 2.6 Behavior experiments

The following behavioral assays were performed in both male and female mice; studies were sufficiently powered to detect sex differences. For clarity of focusing on genotype, data presented in the main body of the text are collapsed across sex. Analyses of sex differences may also be found in supporting information. Detailed descriptions of behavioral assay methodologies may be found in supporting information.

#### 2.6.1 Oxycodone conditioned place preference

Mice underwent a modified protocol of oxycodone conditioned place preference (CPP).^28^ Any mouse that showed an initial side preference greater than 200 s during the 20min pretest was given oxycodone in its initially nonpreferred side. Conditioning sessions (5 min) occurred twice a day for 3 days, with saline (10 ml/kg i.p.) conditioning sessions occurring in the morning (0900) and oxycodone (0.05, 0.5, or 5 mg/kg i.p.) conditioning sessions occurring 4 h later. A day after the final conditioning session, mice underwent a drug‐free 20min test for CPP, comparing the amount of time spent in the oxycodone‐paired and saline‐paired sides.

#### 2.6.2 Open‐field locomotor activity

Open‐field chambers were used to measure baseline (10 ml/kg saline i.p.) and oxycodone‐induced (5 mg/kg i.p.) locomotor activity. Each session was 20 min long.

#### 2.6.3 Naloxone‐precipitated withdrawal

Oxycodone was administered using a modified dose ramping protocol (10 to 40 mg/kg over 8 days) previously demonstrated to produce oxycodone dependency in C57BL/6J mice.^29^ Following development of oxycodone dependency, baseline (10ml/kg saline i.p.) and naloxone‐precipitated (5mg/kg i.p.) opioid withdrawal‐related behaviors were assessed for 10 min: paw shakes, wet dog shakes, jumps, ptosis, body tremor, teeth chattering, piloerection, and diarrhea. A global withdrawal score was calculated to give all withdrawal behaviors proportional weighting, as previously reported.^30^

#### 2.6.4 Nociception/shock flinch

Baseline (saline 10 ml/kg s.c.) and post‐oxycodone (3 mg/kg s.c.) startle responses were measured in response to a variety of shock intensities.

#### 2.6.5 Two‐bottle choice

Oxycodone, sucrose, and quinine consumption and preference were measured using a modified two‐bottle choice (2BC) protocol with self‐made ball‐bearing sipper tubes, similar to those previously described.^31^

#### 2.6.6 Food consumption

The amount of food in the home cage was weighed every 24 h, 11 h into the dark cycle, for 5 days. The amount of food consumed each day was calculated by subtracting the amount of food remaining from the previous day's food weight.

### 2.7 Data analysis

Experimenters were blinded to genotype during all stages of data collection. Data are presented as the mean ± SEM. Data were analyzed using GraphPad Prism 8 (GraphPad, La Jolla, CA, USA). The level of significance was set at *p* < 0.05 for all analyses. Statistically significant individual data point outliers were identified using the ROUT method with *Q* = 1% and excluded. Some data were excluded on the basis of technical errors, such as leaking drinking tubes or equipment failures that occurred during a measurement session. Normal distribution was assessed prior to statistical analysis. Two‐tailed unpaired *t* tests and two‐tailed paired *t* tests were used to analyze normally distributed data. Mann‑Whitney *U* tests were used to analyze non‐normally distributed data. For data with multiple groups and/or repeated measures, ANOVA or restricted maximum likelihood (REML) with Sidak's post hoc tests was used. REML was used if data points were missing due to removal of outliers or experimental exclusion.

## 3 RESULTS

### 3.1 MORflox‐vGluT2cre mice lack MOR expression in vGluT2‐expressing neurons

**FIGURE 1:**
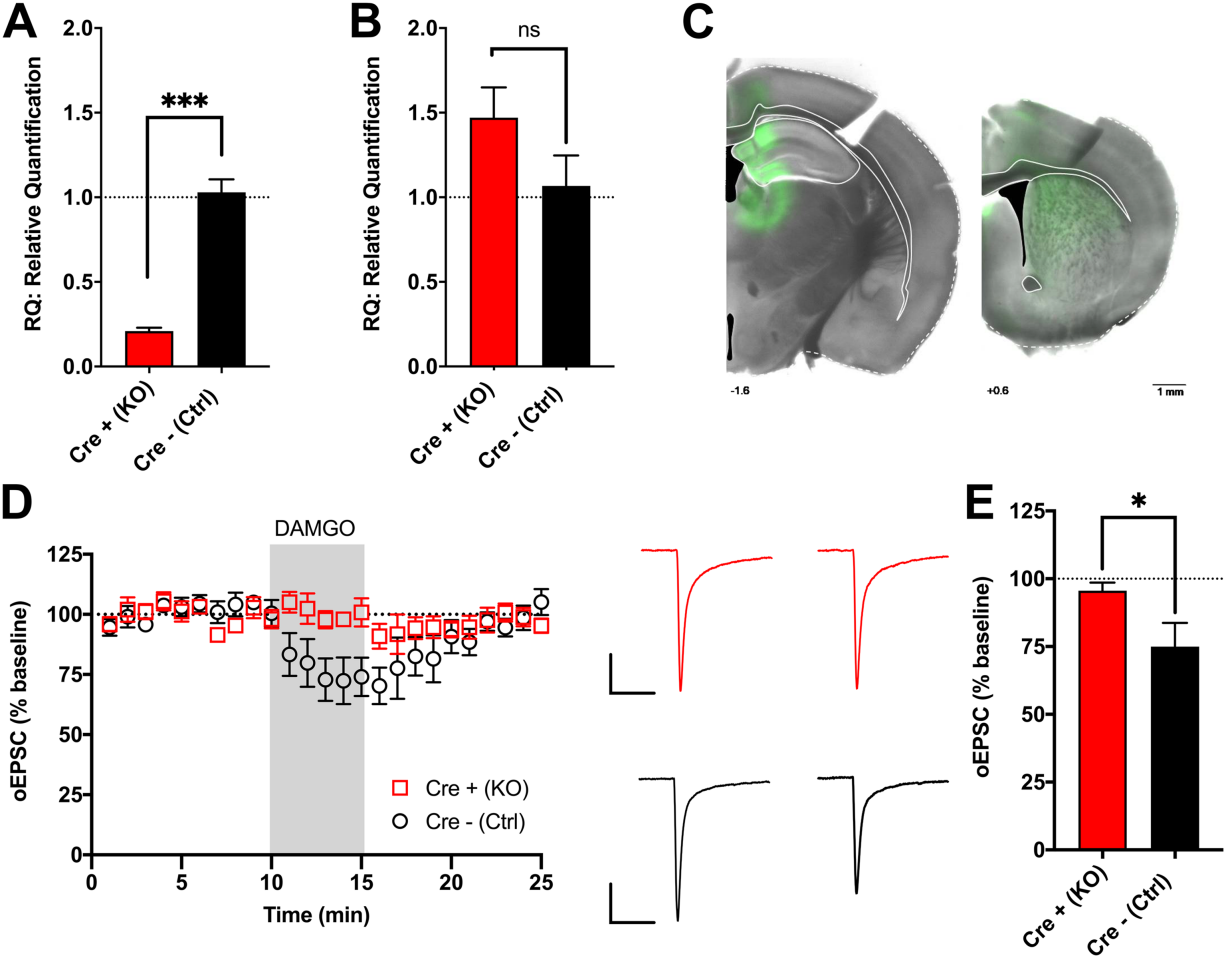
MORflox‐vGluT2cre mice lack MOR‐expression in vGluT2‐expressing neurons. (A) Male Cre+ (KO) animals had less MOR (Oprm1) mRNA expression in the thalamus than Cre− (Ctrl) littermate controls (*n* = 3,3). (B) There were no differences in cortical MOR mRNA expression between male Cre+ (KO) and Cre− (Ctrl) animals (*n* = 3,3). (C) Representative image showing eYFP expression in male Cre+ (KO) mouse injected with AAV.ChR2.eYFP into the thalamus with expression of ChR2 in the terminal fields within dorsal striatum. (D) In male Cre− (Ctrl) mice, the MOR agonist DAMGO (300 nM, 5 min) reduced optically evoked excitatory postsynaptic currents (oESPCs) in response to thalamic input stimulation in dorsal striatal medium spiny neurons; however, DAMGO failed to inhibit glutamate release in male Cre+ (KO) mice (*n* = 6, 5). Also depicted are representative oEPSC traces of baseline (average of 1‑10 min, left) and DAMGO treatment (average of 12‑17 min, right). Scale bars = 100 pA, 50 ms. (E) Following application of DAMGO, male Cre− (Ctrl) mice had decreased average oEPSC magnitudes compared with baseline (12‑17 min vs. baseline); whereas male Cre+ (KO) mice showed no change from baseline. *p < 0.05; ***p < 0.001. Error bars indicate ± SEM. Cre+ (KO) = MORflox‐vGluT2cre positive (+); Cre– (Ctrl) = MORflox‐vGluT2cre negative (−)

To ablate MOR expression specifically within vGluT2‐expressing neurons, we bred conditional MOR knockout mice (MORflox) with mice that express Cre‐recombinase in vGluT2‐expressing neurons (vGluT2cre), similar to previous studies.^21^, ^27^ We assessed Cre‐expressing vGluT2 neuron MOR knockout mice and littermate controls, identified here as Cre+ (KO) and Cre− (Ctrl), respectively. The thalamus is a region with a high density of vGluT2‐expressing neurons.^22^ MOR mRNA expression was significantly decreased in the thalamus of Cre+ (KO) mice, compared with Cre− (Ctrl) controls (Figure 1A, unpaired *t* test: *p* = 0.0005, *t*_4_ = 10.42). MOR mRNA levels were also assessed in the cortex, a region with low vGluT2‐expression.^22^ In cortex, MOR expression was not significantly different between genotypes (Figure 1B, Mann‑Whitney test, *p* = 0.19). To functionally assess the knockout of MORs in vGluT2‐expressing neurons in Cre+ (KO) mice, we investigated a vGluT2‐expressing synapse we had previously shown to exhibit MOR‐mediated inhibition of glutamate transmission.^21^ Our previous work demonstrated that MORs inhibit glutamate transmission at vGluT2‐expressing thalamostriatal synapses but did not reveal if it was specifically MORs within the vGluT2 neurons themselves that mediated that inhibition.^21^ To address this question, as well as to functionally assess MOR knockout, we performed whole cell patch clamp electrophysiological recordings from dorsolateral striatal medium spiny neurons and specifically stimulated vGluT2‐expressing thalamic inputs. To accomplish this, we injected AAV9.hSyn.ChR2(H134R)‐eYFP into the thalamus of Cre− (Ctrl) and Cre+ (KO) animals (Figure 1C) and used optical stimulation to evoke excitatory postsynaptic currents (oEPSCs) in medium spiny neurons. Following application of the MOR agonist, DAMGO (300 nM, 5 min), oEPSCs were decreased in Cre− (Ctrl) but not Cre+ (KO) mice (Figure 1D,E, unpaired *t* test: *p* = 0.04, *t*_9_ = 2.41). Overall, these results are supportive evidence that MORs were deleted from vGluT2‐expressing neurons in Cre+ (KO) mice.

### 3.2 MORflox‐vGluT2cre mice lack oxycodone‐induced conditioned place preference and locomotor stimulation

**FIGURE 2:**
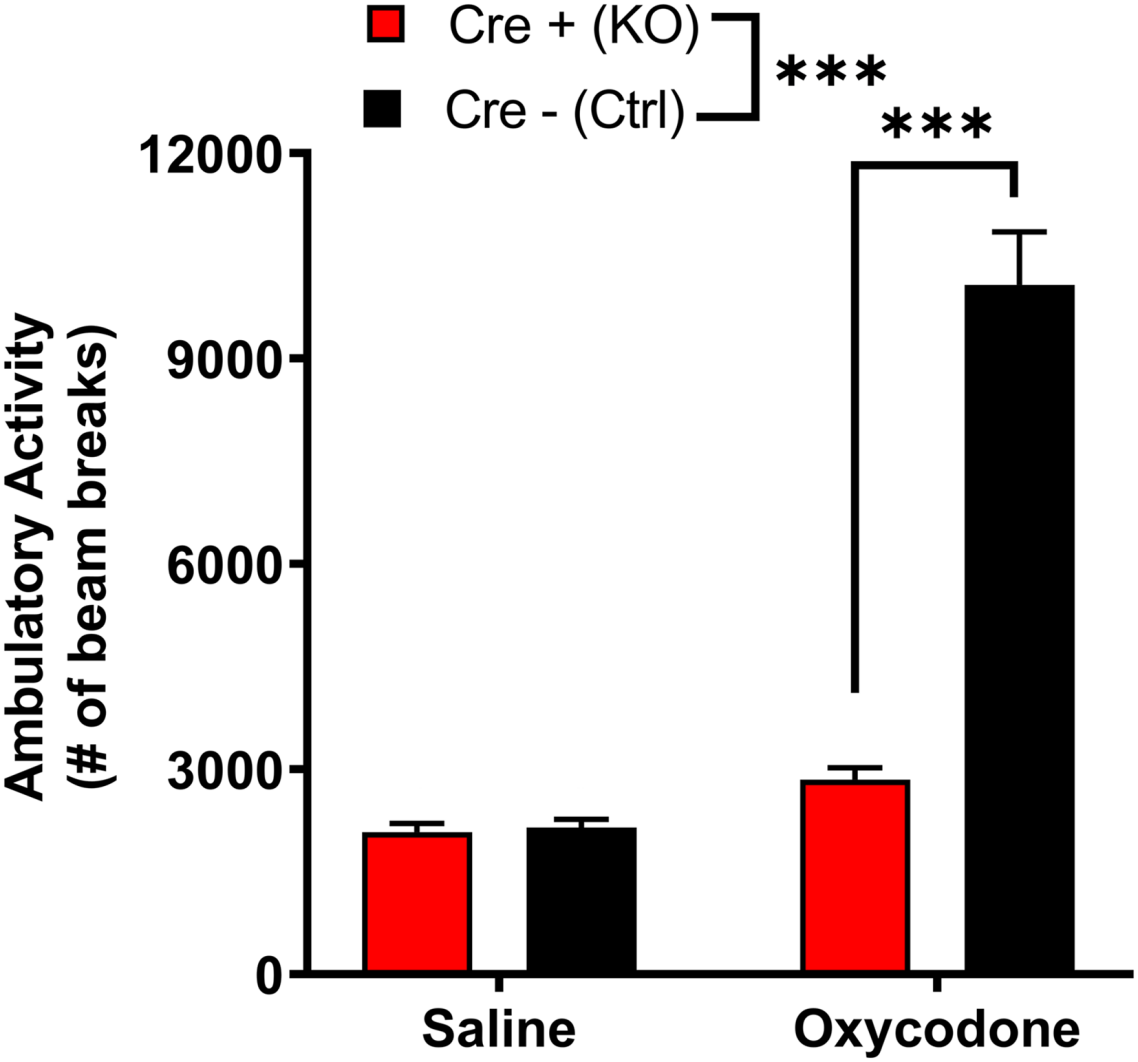
MORflox‐vGluT2cre mice lack oxycodone‐induced locomotor stimulation. There were no baseline differences in locomotor activity in male and female Cre+ (KO) and Cre− (Ctrl) mice, but Cre+ (KO) mice lacked the oxycodone‐induced locomotor stimulation seen in controls [*n* = 17 (8M/8F) Cre+ (KO), 21 (10M/11F) Cre− (Ctrl)]. No sex differences were detected. Data are collapsed across sex. ***p < 0.001. Error bars indicate ±SEM. Cre+ (KO) = MORflox‐vGluT2cre positive (+); Cre– (Ctrl) = MORflox‐vGluT2cre negative (−)

**FIGURE 3:**
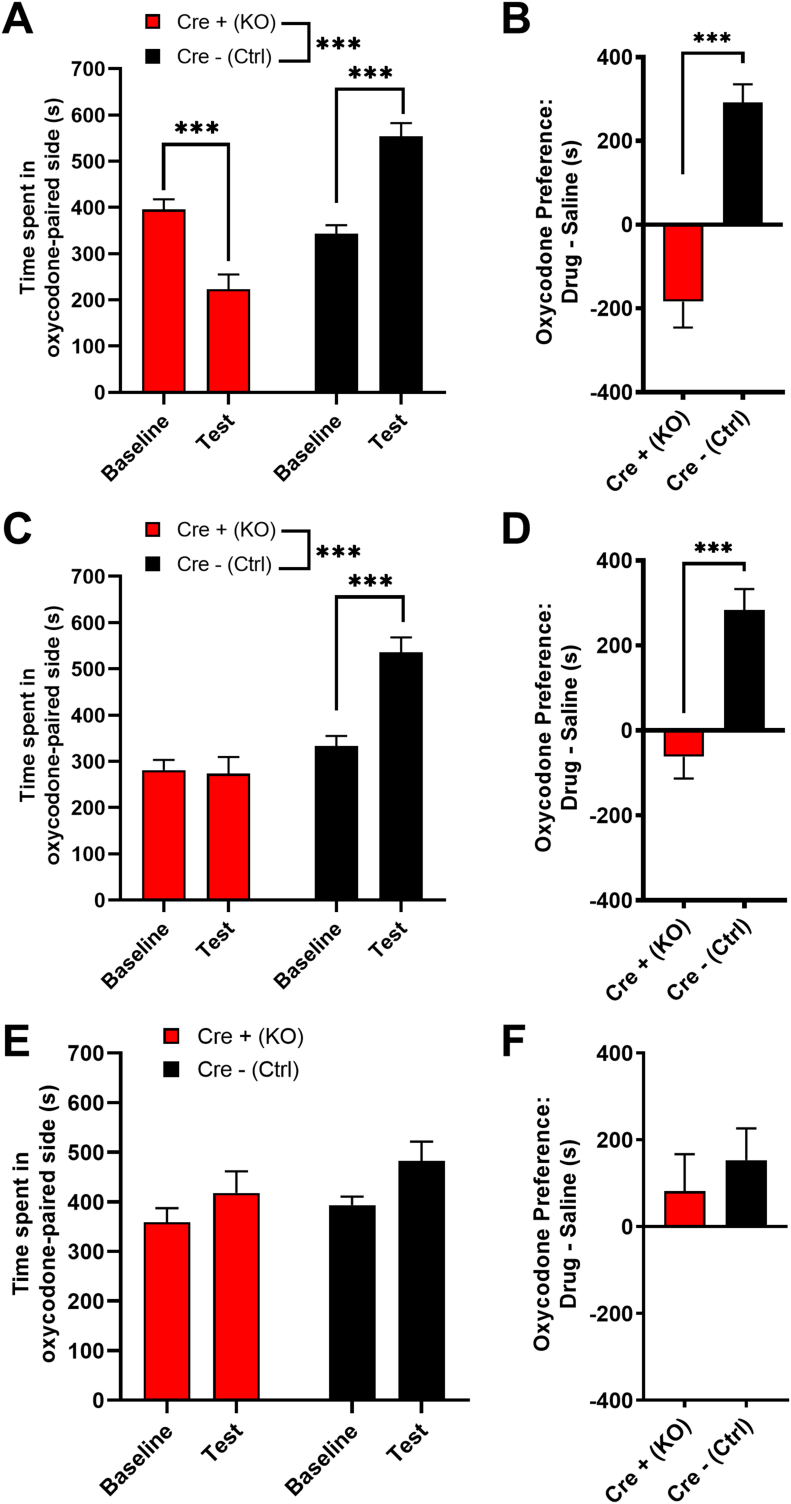
MORflox‐vGluT2cre mice lack oxycodone‐induced conditioned place preference. (A) Following conditioning with 5 mg/kg oxycodone, male and female Cre+ (KO) mice spent less time in the oxycodone‐paired side, whereas Cre− (Ctrl) mice spent more time in the oxycodone‐paired side [*n* = 18 (9M/9F) Cre+ (KO), 17 (9M/8F) Cre− (Ctrl)]. No sex differences were detected. (B) Cre+ (KO) mice showed conditioned place aversion for 5mg/kg oxycodone, whereas Cre− (Ctrl) mice showed conditioned place preference. (C) Following conditioning with 0.5mg/kg oxycodone, Cre− (Ctrl) mice spent more time on the oxycodone‐paired side, whereas Cre+ (KO) did not change the amount of time spent in the oxycodone‐paired side [*n* = 16 (8M/8F) Cre+ (KO), 13 (5M/8F) Cre− (Ctrl)]. No sex differences were detected. (D) Cre− (Ctrl) mice showed conditioned place preference for 0.5mg/kg oxycodone, whereas Cre+ (KO) mice did not. (E) Following conditioning with 0.05mg/kg oxycodone, neither Cre− (Ctrl) nor Cre+ (KO) mice spent significantly more time in the oxycodone‐paired side, although there was a significant overall impact of test [*n*=16 (8M/8F) Cre+ (KO), 14 (7M,7F) Cre− (Ctrl)]. (F) Cre− (Ctrl) and Cre+ (KO) mice did not differ in their conditioned place preference scores for 0.05mg/kg oxycodone. Data are collapsed across sex. ***p < 0.001. Error bars indicate ± SEM. Cre+ (KO) = MORflox‐vGluT2cre positive (+); Cre– (Ctrl) = MORflox‐vGluT2crenegative (−)

MORs are involved in the locomotor and rewarding effects of opioids.^4^ Low doses of opioids produce locomotor stimulation.^32^ Therefore, we examined the locomotor activity of MORflox‐vGluT2cre mice following an injection of either saline (10 ml/kg i.p.) or oxycodone (5 mg/kg i.p.). The two genotypes had differential responses to treatment (Figure 2; two‐way ANOVA: treatment, *p* < 0.0001, F(1,35) = 95.82; genotype, *p* < 0.0001, F(1,35) = 59.71; interaction, *p* < 0.0001, F(1,35) = 64.98). Although there were no differences in basal locomotor activity between Cre+ (KO) and Cre− (Ctrl) mice following saline injection, Cre− (Ctrl) mice had a greater locomotor response to oxycodone than Cre+ (KO) mice (Sidak's test: saline *p* = 0.99; oxycodone *p* < 0.0001). Cre− (Ctrl) mice showed oxycodone‐induced locomotor stimulation (*p* < 0.0001), whereas Cre+ (KO) mice did not (*p* = 0.45). To measure oxycodone reward in these mice, we used conditioned place preference (CPP), a well‐validated measure of drug reward.^33^ Following conditioning with 5mg/kg oxycodone, Cre− (Ctrl) mice spent more time in the oxycodone‐paired side, whereas Cre+ (KO) mice spent less time in the oxycodone‐paired side during the drug‐free test (Figure 3A, two‐way ANOVA: genotype *p* < 0.0001, F(1,33) = 44.86; test *p* = 0.54, F(1,33) = 0.38; interaction *p* < 0.0001, F(1,33) = 39.78; Sidak's test: baseline vs. test, Cre+ (KO) *p* = 0.0005; Cre− (Ctrl) *p* < 0.0001). Assessments of side preference showed that Cre− (Ctrl) mice displayed CPP for the oxycodone‐paired side, whereas Cre+ (KO) mice showed conditioned place aversion (Figure 3B, Mann‑Whitney test, *p* < 0.0001). These data suggest that Cre+ (KO) mice may find oxycodone aversive, unlike Cre− (Ctrl) mice, which find oxycodone rewarding. However, an alternative hypothesis is that Cre+ (KO) mice are more sensitive to the effects of oxycodone and would find a lower dose of oxycodone rewarding; therefore, we tested two lower oxycodone doses (0.5 and 0.05 mg/kg) in separate cohorts of mice. Following conditioning with 0.5mg/kg oxycodone, Cre− (Ctrl) mice spent more time in the oxycodone‐paired side, whereas Cre+ (KO) mice did not change the amount of time spent in the oxycodone‐paired side (Figure 3C, two‐way ANOVA: test, *p* = 0.0023, F(1,27) = 11.28; genotype, *p* < 0.0001, F(1,27) = 28.04; interaction, *p* = 0.0012, F(1,27) = 13.21; Sidak's test: baseline vs. test, Cre+ (KO) *p* = 0.97; Cre− (Ctrl) *p* = 0.0001). Cre− (Ctrl) mice showed CPP for the oxycodone‐paired side, whereas Cre+ (KO) mice did not (Figure 3D, unpaired *t* test: *p* < 0.0001, *t*_27_ = 4.80). Following conditioning with 0.05mg/kg oxycodone, both Cre− (Ctrl) and Cre+ (KO) mice spent slightly more time in the oxycodone‐paired side (Figure 3E, two‐way ANOVA: test, *p* = 0.015, F(1,28) = 6.72; genotype, *p* = 0.22, F(1,28) = 1.59, interaction, *p* = 0.602, F(1,28) = 0.28). While there was a main effect of test, post hoc analyses failed to reveal a significant effect of test for either genotype (Sidak's test: baseline vs. test, Cre+ (KO) p = 0.26; Cre− (Ctrl) p = 0.082), suggesting that both Cre+ (KO) and Cre− (Ctrl) mice acquire little to no oxycodone CPP at this dose. An assessment of CPP score also revealed no genotype difference (Figure 3F, unpaired *t* test: *p* = 0.54, *t*_28_ = 0.62). Altogether, these data suggest that Cre+ (KO) mice are not hypersensitive to the rewarding effects of oxycodone. Because MORs are also involved in mediating the rewarding effects of ethanol,^4^ we hypothesized that Cre+ (KO) mice would also have disrupted ethanol reward. Both Cre+ (KO) and Cre− (Ctrl) mice showed CPP for ethanol (3 g/kg i.p.), suggesting that ethanol reward is intact in Cre+ (KO) mice (Figure S1). These results suggest that MORs on vGluT2‐expressing neurons are involved in both the locomotor stimulating and rewarding effects of oxycodone.

### 3.3 MORflox‐vGluT2cre mice have reduced oral oxycodone consumption

**FIGURE 4:**
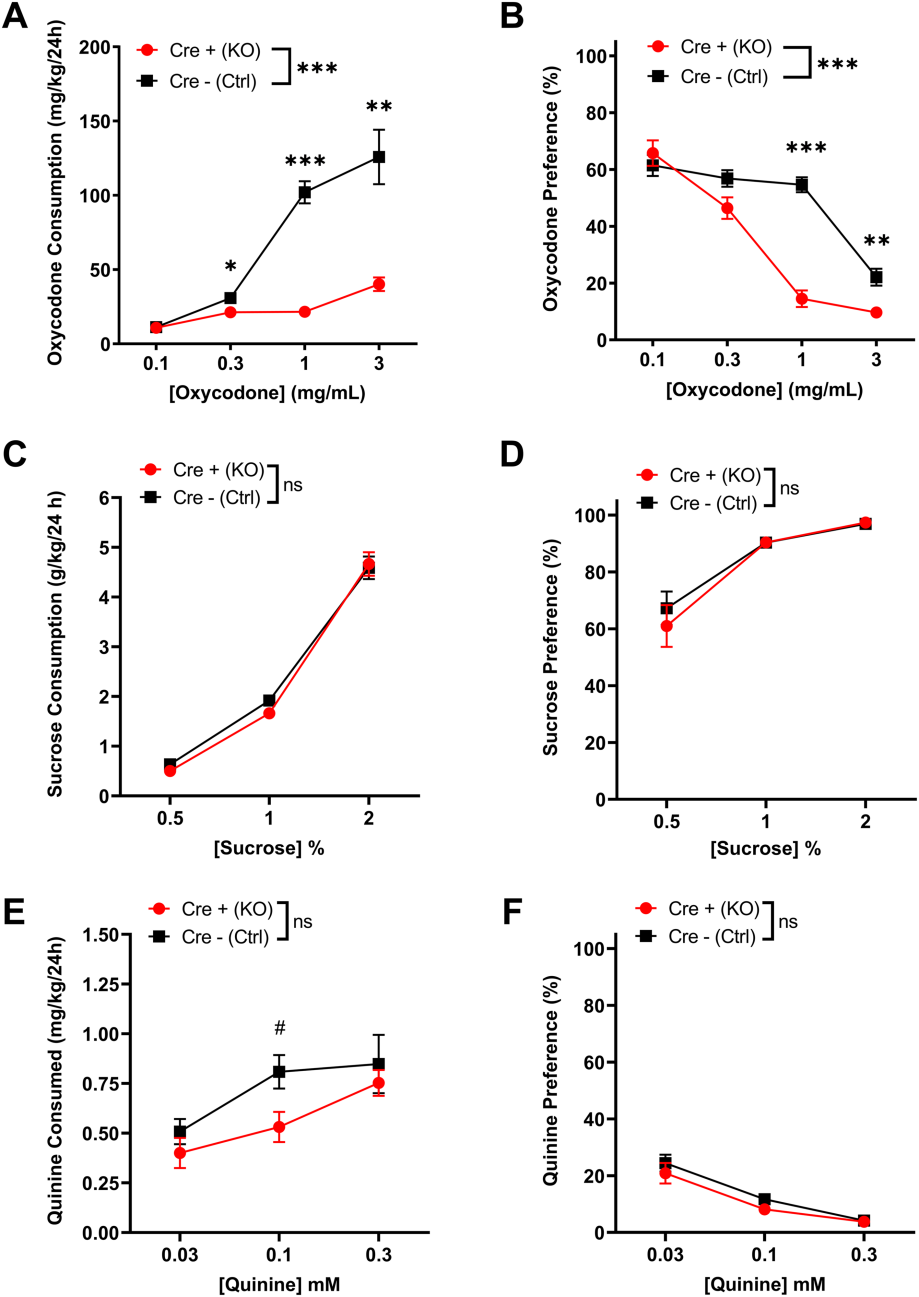
MORflox‐vGluT2cre mice have reduced oral oxycodone consumption but unchanged sucrose and quinine consumption. (A) Cre+ (KO) mice drank less oxycodone solution than Cre− (Ctrl) controls at 0.3, 1, and 3mg/ml concentrations [*n* = 16 (8M/8F) Cre+ (KO); *n* = 17 (9M/8F) Cre− (Ctrl)]. No sex differences were detected. (B) Cre+ (KO) mice showed less preference for oxycodone solution over water than Cre− (Ctrl) controls at 1 and 3mg/ml concentrations. No sex differences were detected. (C) There were no differences in sucrose consumption or (D) preference between genotypes [*n* = 16(9M/7F) Cre+ (KO), 17(8M/9F) Cre− (Ctrl)]. E) There were no statistically significant differences in quinine consumption or (F) preference between genotypes [*n*=16(9M/7F) Cre+ (KO), 17(8M/9F) Cre− (Ctrl)], although there was a trend at 0.1 mM quinine. Data presented here are collapsed across sex for clarity. Findings where sex differences were identified may be found in Figure S4 and Table S1. *p < 0.05; **p < 0.01; ***p < 0.001. ^#^ *p* = 0.06. Error bars indicate ± SEM. Cre+ (KO) = MORfloxvGluT2cre positive (+); Cre– (Ctrl) = MORflox‐vGluT2cre negative (−)

To further characterize oxycodone reward in MORflox‐vGluT2cre mice, we used a 24 h 2BC preference assessment of oral consumption of oxycodone or water. First, we established this method in pilot experiments using 1mg/ml oxycodone solution and C57BL/6J mice and found that these mice readily consumed a 1mg/mL solution of oxycodone (Figure S2). Therefore, we moved forward with testing MORflox‐vGluT2cre mice. However, in order to assess sensitivity to oxycodone, in these experiments, we used a within‐subject assessment of several different concentrations of oxycodone. Cre+ (KO) mice consumed less oxycodone (Figure 4A **,** two‐way ANOVA: genotype *p* < 0.0001, F(1,31) = 40.83; concentration *p* < 0.0001, F(1.29, 40.07) = 47.95; interaction *p* < 0.0001, F(3, 93) = 22.79; Sidak's test: 0.1 mg/ml, *p* = 0.99; 0.3 mg/ml, *p* = 0.020; 1 mg/ml, *p* < 0.001; 3 mg/ml, *p* = 0.0011) and showed decreased preference for oxycodone compared with Cre− (Ctrl) mice (Figure 4B, two‐way ANOVA: genotype *p* = 0.00020, F(1,31) = 18.19; concentration *p* < 0.0001, F(2.62, 81.08) = 141.30; interaction *p* < 0.0001, F(3, 93) = 28.08; Sidak's test: 0.1 mg/ml, *p* = 0.92; 0.3 mg/ml, *p* = 0.14; 1 mg/ml *p* < 0.0001; 3 mg/ml *p* = 0.0056). Cre− (Ctrl) control mice showed decreased preference for 3mg/ml oxycodone concentration compared with other concentrations (Sidak's test: 0.1 vs. 3: *p* < 0.0001; 0.3 vs. 3: *p* < 0.0001; 1 vs. 3: *p* < 0.0001). Cre+ (KO) mice displayed significantly decreasing preferences for each higher concentration, except between 1 and 3 mg/ml (Sidak's test: 0.1 vs. 0.3, *p* = 0.0003; 0.1 vs. 1, *p* < 0.0001; 0.1 vs. 3, *p* < 0.0001; 0.3 vs. 1, *p* < 0.0001; 0.3 vs. 3, *p* < 0.0001; 1 vs. 3: *p* = 0.4982). There were no sex differences in oxycodone consumption or preference (Figure S4A,B; Table S1). Total fluid consumption did not significantly differ between genotypes (Figure S3A). Females drank significantly more fluid overall than males, and there were significant differences in the amount of fluid consumed at each of the concentrations (Figure S4C; Table S1). Altogether, these data suggest that Cre+ (KO) mice do not find oxycodone as rewarding as Cre− (Ctrl) mice; however, an alternative hypothesis is that these mutant mice have altered preferences for rewarding substances or are possibly more sensitive to the bitter taste of oxycodone. Therefore, a separate group of mice underwent two series of 2BC: one with escalating concentrations of sucrose (0.5%, 1%, and 2% w/v), a naturally rewarding substance, and the other with escalating concentrations of quinine (0.03, 0.1, and 0.3 mM), a naturally aversive, bitter substance. In both series, the preference was tested against water and mice were counterbalanced in which order they underwent each series with a 1week washout period between each series. There were no genotype differences in sucrose consumption (Figure 4C; two‐way ANOVA: genotype, *p* = 0.48, F(1,31) = 0.52; concentration, *p* < 0.0001, F(1.163, 36.06) = 526.60; interaction, *p* =0 .43, F(2, 62) = 0.86), preference (Figure 4D; two‐way ANOVA: genotype, *p* = 0.58, F(1,31) = 0.32; concentration, *p* < 0.0001, F(1.036, 32.10) = 43.04; interaction, *p* = 0.62, F(2, 62) = 0.49), or total volume of fluid consumed during sucrose dinking sessions (Figure S3B), although there were significant effects of sex (Figure S4D‑F; Table S1). There were also no genotype differences in quinine consumption (Figure 4E; two‐way ANOVA genotype, *p* = 0.090, F(1,31) = 3.069; concentration, *p* = 0.0006, F(1.591, 49.31) = 9.87; interaction, *p* = 0.44, F(2, 62) = 0.84), preference (Figure 4F; two‐way ANOVA: genotype, *p* = 0.22, F(1,31) = 1.60; concentration, *p* < 0.0001, F(1.149, 35.62) = 53.61; interaction, *p* = 0.62, F(2, 62) = 0.48), or total volume of fluid consumed during quinine dinking sessions (Figure S3C). There were significant effects of sex on total fluid consumption during quinine drinking (Figure S4G‑I; Table S1). Cre+ (KO) and Cre− (Ctrl) mice also did not differ in food consumption, but there was a sex difference detected (Figure S5A). Baseline weights were recorded for all mice involved in behavior experiments, before beginning testing. Cre+ (KO) mice weighed slightly less than Cre− (Ctrl) mice (Figure S5B). Altogether, these results indicate that the genotype differences in oral oxycodone consumption are not due to general differences in sensitivity to naturally rewarding or aversive substances and further suggest that MORs on vGluT2Cre−expressing neurons are specifically involved with opioid reward.

### 3.4 MORflox‐vGluT2cre mice have intact oxycodone‐induced antinociception

**FIGURE 5:**
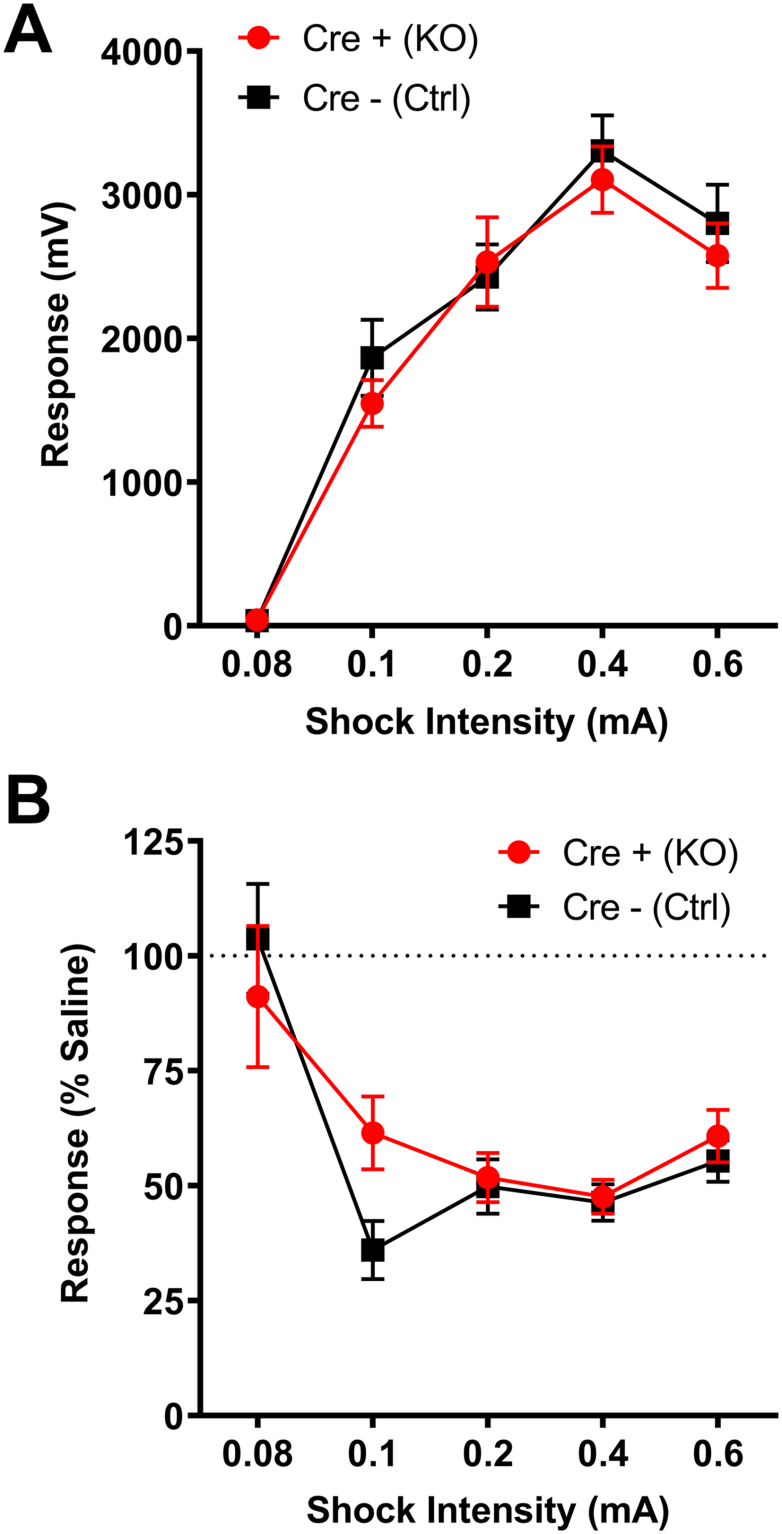
MORflox‐vGluT2cre mice have intact oxycodone‐induced antinociception. (A) Cre+ (KO) and Cre− (Ctrl) mice have equivalent nociceptive responses to a range of shock stimuli mice [*n* = 17 (9M/8F) Cre+ (KO), 18(8M/10F) Cre− (Ctrl)]. (B) A 3mg/kg oxycodone treatment produces equivalent antinociception in both Cre+ (KO) and Cre− (Ctrl) mice, although there was a trend at the 0.1mA shock intensity for Cre+ (KO) mice to be less sensitive to oxycodone treatment (*p* = 0.099). Data presented here are collapsed across sex for clarity. Sex differences are presented in Figure S6. Error bars indicate ± SEM. Cre+ (KO) = MORflox‐vGluT2cre positive (+); Cre– (Ctrl) = MORflox‐vGluT2cre negative (−). mV = millivolts

Because MORs are also involved in antinociception,^4^ we measured responses to painful stimuli using the shock‐flinch test. We used this method to measure antinociception because the stimulating‐ and straub tail‐inducing effects of oxycodone cause it to be difficult to obtain accurate and reliable measurements from traditional pain assessment tests, such as the hot plate, tail flick, and Hargreaves methodologies. There were no differences in baseline (saline 10 ml/kg s.c.) responses to shocks of various intensities between genotypes (Figure 5A; residual maximum likelihood analysis: genotype, *p* = 0.53, F(1,34) = 0.41; shock intensity, *p* < 0.0001, F(3.27,105.50) = 79.88; interaction, *p* = 0.79, F(4,129) = 0.43). Male mice were significantly more sensitive to shock treatments (Figure S6A). Following an oxycodone injection (3 mg/kg s.c.), responses to shocks decreased equally in both male and female Cre− (Ctrl) and Cre+ (KO) animals (Figures S5B and S6B; residual maximum likelihood analysis: genotype, *p* = 0.3508, F(1,34) = 0.90; shock intensity, *p* < 0.0001, F(4,132) = 17.41; interaction, *p* = 0.054, F(4,132) = 2.39). These results indicate that pain responses and oxycodone‐induced antinociception are intact in Cre+ (KO) animals, suggesting that MORs on vGluT2‐expressing neurons are not involved in nociception or the antinociceptive effect of oxycodone.

### 3.5 MORflox‐vGluT2cre mice show baseline oxycodone withdrawal‐like responses and no increase in withdrawal responses following naloxone treatment

**FIGURE 6:**
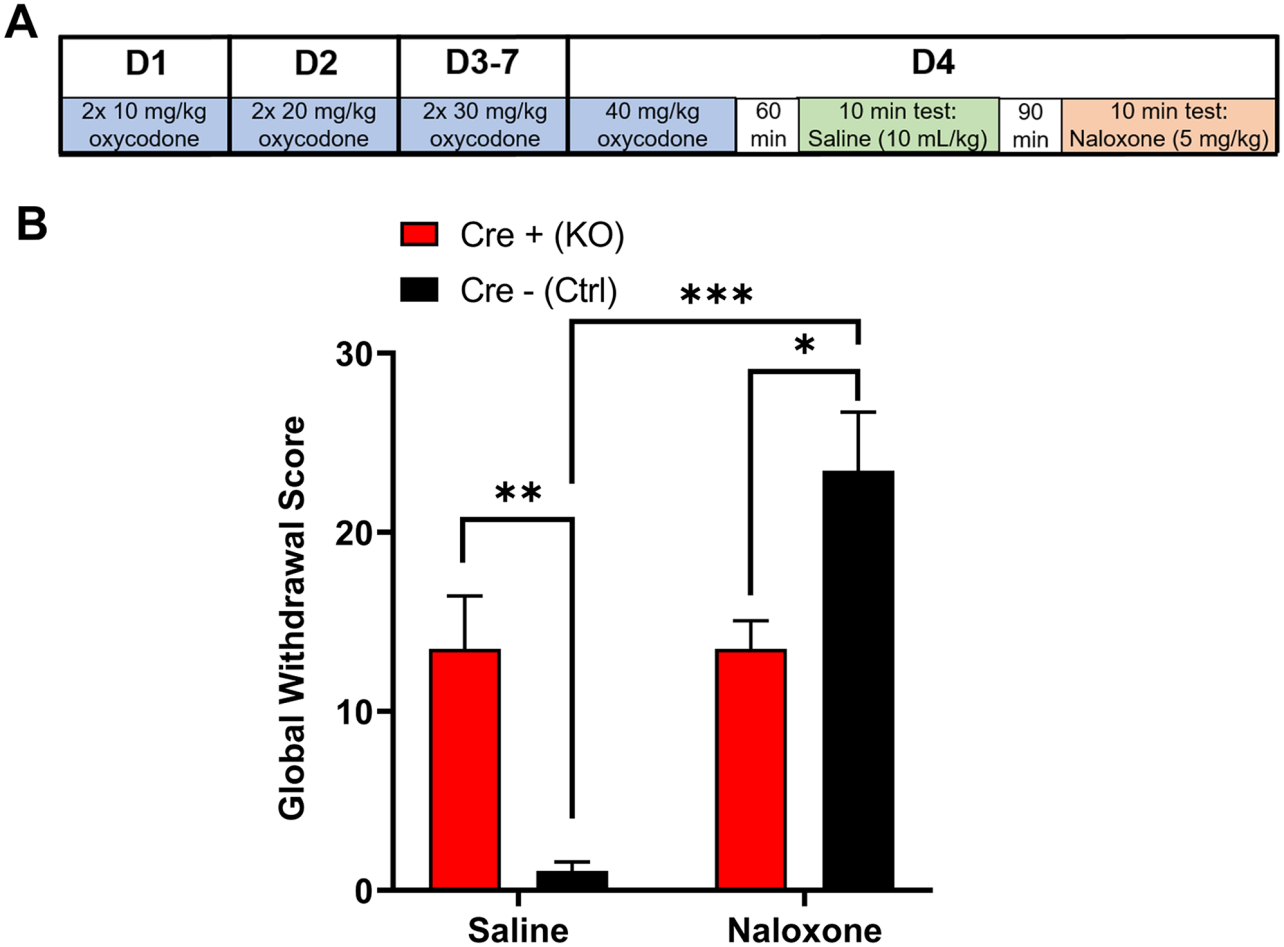
MORflox‐vGluT2cre mice have altered oxycodone withdrawal‐related behaviors. (A) Schematic of experiment schedule. (B) Global withdrawal scores for mice following a saline injection and 1.5 h later, a naloxone injection [(*n* = 16(8M/8F) Cre+ (KO),13(5M/8F) Cre− (Ctrl)]. Cre+ (KO) mice had a higher global withdrawal score following saline injection than Cre− (Ctrl) mice but had a lower global withdrawal score following naloxone injection than Cre− (Ctrl) mice. Data are collapsed across sex. Data for individual withdrawal‐related behaviors are presented in Figure S7. *p < 0.05; **p < 0.01; ***p < 0.001. Error bars indicate ± SEM. Cre+ (KO) = MORflox‐vGluT2cre positive (+); Cre– (Ctrl) = MORflox‐vGluT2cre negative (−)

To determine if Cre+ (KO) mice differ from Cre− (Ctrl) mice in the development of oxycodone dependency or naloxone‐precipitated withdrawal, we used a dose‐ramping schedule followed by an assessment of baseline opioid withdrawal‐like behaviors (saline, 10 ml/kg i.p.) and a subsequent assessment after a naloxone injection (5 mg/kg i.p.; Figure 6A). Assessment of a global withdrawal score revealed a main effect of treatment, but not genotype (two‐way ANOVA: genotype, *p* = 0.64, F(1,27) = 0.23; treatment, *p* < 0.0001, F(1,27) = 25.90), although there was a significant interaction between treatment and genotype (*p* < 0.0001, F(1,27) = 25.93; Figure 6B). Surprisingly, following saline injection, Cre+ (KO) mice had a higher global withdrawal score than Cre− (Ctrl) mice (Sidak's test: saline, *p* = 0.0093). Following naloxone injection, there was an increase in the global withdrawal score of Cre− (Ctrl) mice, but there was no change in Cre+ (KO) mice (Sidak's test: Cre+ (KO), *p* > 0.99; Cre− (Ctrl) *p* < 0.0001). Additionally, the global withdrawal score following naloxone‐precipitated withdrawal was higher for Cre− (Ctrl) mice than Cre+ (KO) mice (Sidak's test: *p* = 0.011). Measures of the individual withdrawal behaviors are displayed in Figure S7. These data indicate that Cre+ (KO) mice show responses typically associated with opioid withdrawal, an hour following an oxycodone injection, before naloxone‐precipitated withdrawal, suggesting that MORs on vGluT2‐expressing neurons are involved in opioid withdrawal‐related behaviors.

## 4 DISCUSSION

The major finding of this study is that MORs found on a subset of glutamatergic neurons that express the glutamate transporter, vGluT2, modulate opioid reward. In the absence of these vGluT2 neuron MORs, oxycodone reward is ablated. In addition, these vGluT2 neuron MORs mediate the locomotor stimulatory effect of opioid treatment, a behavioral response common to many drugs of abuse,^34^ as well as opioidwithdrawal‐related behaviors. Many studies find that MOR‐mediated regulation of GABA transmission is critical for opioid reward, opioid‐induced locomotion, and opioid self‐administration.^5^ The current data therefore suggest that there may be complex interplay between MOR‐mediated regulation of GABA and glutamate transmission. Although MOR expression has been documented in glutamatergic neurons and MOR‐mediated regulation of glutamate transmission has been reported for many brain regions, our findings demonstrate a role for MORs expressed in a specific subpopulation of glutamate neurons in modulating opioid reward.^8^, ^9^, ^10^, ^11^, ^12^, ^13^, ^14^, ^15^

Total MOR KO mice lack opioid‐induced locomotor stimulation, opioid CPP, opioid withdrawal symptoms following naloxone‐precipitated withdrawal, and morphine‐induced antinociception.^4^ Similar to the total MOR KO, our Cre+ (KO) mice also lack opioid‐induced locomotor stimulation, have reduced opioid consumption, and show little to no CPP for low doses of oxycodone (≤0.5 mg/kg). However, the Cre+ (KO) mice show conditioned place aversion, rather than a lack of preference, for a higher dose of oxycodone (5 mg/kg) and oxycodone‐induced antinociception remains intact in these animals. The Cre+ (KO) mice do not have a lack of opioid withdrawal‐like behaviors like the total KO mice; instead, the Cre+ (KO) mice display withdrawal‐like responses following the induction of oxycodone dependence, prior to naloxone‐precipitated withdrawal. These data could mean that MORs on vGluT2‐expressing neurons may specifically modulate certain withdrawal responses. It is possible Cre+ (KO) mice experience a negative affective state in response to high doses of oxycodone, which may partially explain why these mice acquire conditioned place aversion for 5mg/kg oxycodone. Alternatively, these mice could experience withdrawal at a faster rate than Cre− (Ctrl) mice. In total MOR KO mice, ethanol CPP is disrupted, whereas ethanol CPP is intact in Cre+ (KO) mice, suggesting that MORs in vGluT2 neurons do not mediate ethanol reward.^4^, ^35^ These data also suggest the behavioral effects of MOR genetic deletion from vGluT2‐expressing neurons is specific to opioids, although additional drugs of abuse should be tested in the future. Food and sucrose consumption are also not affected in Cre+ (KO) mice and they weigh less than their Cre− (Ctrl) littermates, whereas total MOR KO mice have altered palatable food intake and show increased body weight.^6^, ^36^, ^37^, ^38^ In forebrain GABAergic neuron‐specific MOR KO mice, opioid‐induced locomotor stimulation was ablated, similar to our Cre+ (KO) mice.^6^ However, the GABA neuron MOR KO mice had increased opioid self‐administration, intact opioid CPP, and reduced ethanol CPP, which all contrast with our results here.^6^, ^35^ A recent study also assessed opioid reward and aversion using mutant mice that lack MOR expression in the medial habenula. In these mice, behavioral responses to morphine were intact, but naloxone‐induced withdrawal‐related aversion was disrupted, further contrasting with our findings.^39^ Altogether, our results suggest that MORs in vGluT2‐expressing neurons have a critical role in modulating opioid reward.

One of the specific challenges moving forward is the identification of the specific vGluT2 neuron MORs that mediate opioid reward. vGluT2 is expressed in primarily glutamatergic neurons and is predominantly expressed in the thalamus, amygdala, hypothalamus, cerebellum, and brainstem.^22^ vGluT2 is also expressed in subpopulations of neurons in the VTA, ventral pallidum (VP), and certain regions of cortex.^22^, ^40^ Activation of vGluT2‐expressing neurons within the VTA causes place preference, suggesting a role for vGluT2 VTA neurons in reward.^26^ VTA vGluT2 neurons also play a role in aversion signaling, given that a subset of VTA vGluT2 neurons responds to aversive stimuli.^24^ Studies have shown that vGluT2 VTA neurons drive aversion through local input to GABAergic interneurons in the VTA, as well as projections to the lateral habenula (LHb).^41^, ^42^ MORs regulate both GABA and glutamate transmission in LHb, suggesting this may be a potential site of MOR action in our behavioral effects.^12^ Activation of vGluT2 VP neurons is aversive; these neurons innervate the LHb and rostromedial tegmental nucleus, as well as VTA GABA and dopamine neurons.^23^, ^25^ MORs in VP regulate behavioral responses to aversive stimuli, however it is important to note that our Cre+ (KO) mice do not show a difference in behavioral responses to quinine, an aversive substance.^43^ However, others have shown that blockade of VP MORs induces morphine conditioned place aversion.^44^ vGluT2 neurons in the lateral hypothalamus that project to VTA dopamine neurons, specifically those dopamine neurons that project to the ventral NAc medial shell, are involved in encoding aversive stimuli.^45^ It is possible that MORs in these aversion processing vGluT2‐expressing brain regions mediate opioid reward by inhibiting these aversion‐encoding pathways. Glutamatergic projections from the rostral intralaminar thalamus to the dorsal striatum are involved in modulating reward through striatal dopamine release and we have both here and previously demonstrated that these synapses also express MORs.^21^, ^46^ It is possible that MORs regulate any of these sets of glutamatergic neurons to produce our observed behaviors and much work remains to be done.

It is important to note that while our intent was to study the behavioral role of MORs in glutamatergic neurons, our study is limited by the fact that vGluT2 is co‐expressed in neurons that are known to release other neurotransmitters, that would not normally be considered classical “glutamatergic” neurons. For example, vGluT2 is expressed in VTA dopamine neurons, which co‐release dopamine and glutamate; however, this represents a small population of vGluT2 neurons in the VTA.^47^ In the brainstem, vGluT2 is expressed in catecholaminergic neurons.^48^ vGluT2 is also expressed in cholinergic spinal cord motor neurons, which synapse onto muscles and cause them to contract.^49^ In addition, vGluT2 can be found in GABAergic neurons in the anteroventral periventricular nucleus; a brain region mainly involved in sex‐specific physiology and behaviors.^50^ Given that most of these brain regions or neuronal subtypes are not part of what are usually considered reward neurocircuits, we do not think these are the likely sites of our behavioral effects. However, it is nonetheless possible that MORs in any one of these neural populations could mediate the effects seen in Cre+ (KO) mice. Future work is needed to determine which vGluT2‐expressing neurocircuits are regulated by MORs and how MORs in these neurocircuits mediate opioid reward.

In conclusion, these MORflox‐vGluT2cre mice are a valuable tool to dissect the role of MORs in a subset of glutamatergic neurons that express vGluT2. Our findings challenge the concept that MORs in GABA neurons are the principle drivers of opioid reward; although, they do not suggest that MORs in GABA neurons are not critical. We have demonstrated that there are vGluT2‐containing neurocircuits that are involved in opioid reward and MORs expressed in these neurons mediate this reward. Although these mice have helped to reveal the role of these MORs in this class of glutamatergic neuron, additional work is needed to isolate the specific vGluT2 populations and brain regions responsible for these effects. Although this task will be challenging, it may ultimately lead to novel combinatorial pharmacological therapeutics for treating opioid abuse and addiction.

## AUTHOR CONTRIBUTIONS

KCR, MJK, GGG, BM, BMF, FY, YG, HJH, and DLH performed experiments and contributed to data analyses. KCR, MJK, GGG, BM, BMF, FY, DLH, and BKA designed experiments, discussed the results, and contributed to all stages of manuscript preparation and editing.

## Supporting information


**Figure S1.** **MORflox‐vGluT2cre mice acquire ethanol CPP.** Ethanol (EtOH) CPP was performed as previously described for oxycodone CPP, except there were 4 conditioning sessions for saline and ethanol (3 g/kg i.p.). **A)** Following conditioning with 3 g/kg ethanol, Cre− (Ctrl) and Cre+ (KO) mice spent more time in the EtOH‐paired side (two‐way ANOVA: Genotype, p=0.86, F (1, 28) = 0.033, Session: p=0.0002, F (1, 28) = 19.07, Interaction, p=0.28, F (1, 28) = 1.24) (Sidak's post hoc tests. Session: Cre+ (KO) p=0.031, Cre− (Ctrl), p=0.0029). n=18 Cre+ (KO), 12 Cre− (Ctrl). **B)** Cre− (Ctrl) and Cre+ (KO) mice showed conditioned place preference for 3 g/kg EtOH. (Unpaired t‐test: p=0.76, t=0.30, df=28). Data collapsed across sex. ***p<0.001. Error bars indicate +/‐ SEM. Cre+ (KO)= MORflox‐vGluT2cre positive (+); Cre– (Ctrl)= MORflox‐vGluT2cre negative (‐).
**S2. C57BL/6J mice orally consume oxycodone. A)** Adult male C57Bl/6J mice orally consumed oxycodone (1 mg/mL dissolved in reverse osmosis water) over twelve 24 hr sessions (n=8). **B)** C57BL/6J mice equally preferred oxycodone and water (one‐sample t‐test, theoretical mean=50, p=0.84, t=0.21, df=6). Error bars indicate +/‐ SEM.
**Figure S3** Associated with Figure 4. **A)** There were no differences between genotypes in total fluid consumption during oxycodone drinking sessions (two‐way ANOVA: Genotype, p=0.16, F (1, 31) = 2.088, Concentration, p=0.032, F (1.582, 49.03) = 4.052, Interaction, p=0.17, F (3, 93) = 1.732). n=16 (8M/8F) Cre+ (KO); n=17 (9M/8F) Cre− (Ctrl). **B)** There were no genotype differences in sucrose consumption, although there was a significant genotype x sucrose concentration interaction (two‐way ANOVA: Genotype, p=0.22, F (1, 31) = 1.55; Concentration, p<0.0001, F (1.751, 54.27) = 69.14; Concentration x Genotype, p=0.0083, F (2, 62) = 5.18) (Sidak's post hoc tests. Genotype: 0.5% p=0.25; 1% p=0.071; 2% p=0.99). n=16(9M/7F) Cre+ (KO), 17(8M/9F) Cre− (Ctrl). **C)** There were no differences between genotypes in fluid consumption during quinine drinking sessions (two‐way ANOVA: Genotype, p=0.39, F (1, 31) = 0.78, Concentration, p=0.32, F (1.93, 59.84) = 1.14, Concentration x Genotype, p=0.030, F (2, 62) = 3.72). n=16(9M/7F) Cre+ (KO), 17(8M/9F) Cre− (Ctrl). Data collapsed across sex. Error bars indicate +/‐ SEM. Cre+ (KO)= MORflox‐vGluT2cre positive (+); Cre– (Ctrl)= MORflox‐vGluT2cre negative (‐).
**Figure S4.** Associated with Figure 4 and Supplemental Table 1. **A‐C)** Assessments of sex and genotype differences in oral oxycodone consumption. n=16 (8M/8F) Cre+ (KO); n=17 (9M/8F) Cre− (Ctrl). Females consumed significantly more fluid than males. **D‐F)** Assessments of sex and genotype differences in oral sucrose consumption. n=16(9M/7F) Cre+ (KO), 17(8M/9F) Cre− (Ctrl). Females consumed significantly more sucrose and total fluid than males but had lower preference for sucrose than males. **G‐I)** Assessments of sex and genotype differences in oral quinine consumption. n=16(9M/7F) Cre+ (KO), 17(8M/9F) Cre− (Ctrl). Females consumed significantly more fluid than males. *p<0.05, **p<0.01, ***p<0.001. Error bars indicate +/‐ SEM. Cre+ (KO)= MORflox‐vGluT2cre positive (+); Cre– (Ctrl)= MORflox‐vGluT2cre negative (‐).
Figure S5
**A)** Food consumption between Cre+ (KO) and Cre− (Ctrl) mice. Female mice of both genotypes consumed more food than males (two‐way ANOVA: Genotype, p=0.46, F (1, 33) = 0.57; Sex, p<0.0001, F (1, 33) = 60.46; Interaction, p=0.56, F (1, 33) = 0.35) (Sidak's post hoc tests. Genotype: Female p=0.56, Male p=0.99; Sex: Cre+ (KO) p<0.0001, Cre− (Ctrl) p<0.0001). n=16 (8M/8F) Cre +(KO), 21 (9M/12F) Cre− (Ctrl) mice. **B)** Cre+ (KO) mice weighed less at baseline (~8 wks old) than Cre− (Ctrl) mice. Females weighed less than males in both genotypes (two‐way ANOVA: Genotype, p<0.0001, F (1, 277) = 21.21; Sex, p<0.0001, F (1, 277) = 938.9; Interaction, p=0.58, F (1, 277) = 0.32) (Sidak's post hoc tests. Genotype: Female p=0.0095, Male p=0.0006; Sex: Cre+ (KO) p<0.0001, Cre− (Ctrl) p<0.0001). n=139 (70M/69F) Cre+ (KO), 142 (69M/73F) Cre− (Ctrl). ***p<0.001. Error bars indicate +/‐ SEM. Cre+ (KO)= MORflox‐vGluT2cre positive (+); Cre– (Ctrl)= MORflox‐vGluT2cre negative (‐).
**Figure S6. Male mice are more sensitive to shock stimuli than female mice.** Associated with Figure 5. n=17 (9M/8F) Cre+ (KO), 18(8M/10F) Cre− (Ctrl) for all panels. **A)** There are no genotype differences in baseline responses to shock stimuli, but male mice are more sensitive to shock stimuli than female mice (Residual maximum likelihood analysis: Shock intensity, p<0.0001, F(4,36)=83.9, Sex, p=0.0019, F(1,9)=18.91, Genotype, p=0.36, F(1,9)=0.92, Shock intensity x Sex, p=0.0034, F(4,36)=4.77, Shock intensity x Genotype, p=0.79, F(4,36)=0.42, Sex x Genotype, p=0.57, F(1,9)=0.36, Shock intensity x Sex x Genotype, p=0.45, F(4,10)=1.0). **B)** Behavioral responses to oxycodone treatment were not impacted by the sex or genotype of the mice. There was a significant effect of shock intensity. (Residual maximum likelihood analysis: Shock Intensity, p<0.0001, F (2.095, 65.46) = 16.19, Sex, p=0.2863, F (1, 32) = 1.176, Genotype, p=0.3947, F (1, 32) = 0.7444, Shock Intensity x Sex, p=0.1994, F (4, 125) = 1.523, Shock Intensity x Genotype, p=0.1981, F (4, 125) = 1.528, Sex x Genotype, p=0.7041, F (1, 32) = 0.1468, Shock Intensity x Sex x Genotype, p=0.8821, F (4, 125) = 0.2930). **p<0.01. Error bars indicate +/‐ SEM. Cre+ (KO)= MORflox‐vGluT2cre positive (+); Cre– (Ctrl)= MORflox‐vGluT2cre negative (‐).
**Figure S7. Individual oxycodone withdrawal‐related behavioral assessments.** Associated with Figure 6. n=16(8M/8F) Cre+ (KO), 13(5M/8F) Cre− (Ctrl) for all panels. **A)** Naloxone treatment produced an increase in wet dog shakes in oxycodone‐dependent Cre− (Ctrl) mice, but not in Cre+ (KO) mice. (two‐way ANOVA: Genotype, p=0.1057, F (1, 27) = 2.803; Treatment, p=0.0028, F (1, 27) = 10.80; Treatment x Genotype, p=0.2621, F (1, 27) = 1.312) (Sidak's post‐hoc, Treatment: Cre+ (KO), p=0.23; Cre− (Ctrl), p=0.012). **B)** Oxycodone‐dependent Cre+ (KO) mice displayed more paw shakes than Cre− (Ctrl) following saline treatment, but naloxone treatment reduced these. (two‐way ANOVA: Genotype, p=0.0034, F (1, 27) = 10.33; Treatment, p=0.0457F (1, 27) = 4.390; Treatment x Genotype, p=0.0004, F (1, 27) = 16.54) (Sidak's post hoc tests. Genotype: Saline p<0.0001, Naloxone p=0.91; Treatment: Cre+ (KO) p=0.0002, Cre− (Ctrl) p=0.35). **C)** Naloxone treatment increased jumping behavior in oxycodone‐dependent Cre− (Ctrl) mice, but not in Cre+ (KO) mice. (two‐way ANOVA: Genotype, p=0.0009, F (1, 27) = 14.05; Treatment, p=0.0003, F (1, 27) = 17.61; Treatment x Genotype, p=0.0009, F (1, 27) = 14.05) (Sidak's post‐hoc tests. Genotype: Saline p>0.9999, Naloxone p<0.0001; Treatment, Cre + (KO) p=0.93, Cre– (Ctrl) p<0.0001). **D)** Oxycodone‐dependency did not result in differences in weight loss between genotypes. (unpaired t‐test, p=0.74, t=0.3425, df=27). No sex differences were detected. Data are collapsed across sex. *p<0.05. Error bars indicate +/‐ SEM. Cre+ (KO)= MORflox‐vGluT2cre positive (+); Cre– (Ctrl)= MORflox‐vGluT2cre negative (‐).
**Table S1. Statistical Analyses of Sex Differences in Oral Consumption of Oxycodone, Sucrose, and Quinine.** Associated with Figure 4 and Supplemental Figure 4. Assessments of consumption and preference (Pref) of each substance and total fluid intake associated with each substance. All data analyzed with three‐way ANOVA with genotype, sex, and substance concentration as factors. Significant differences are marked in shaded cells.
