# Supplementary material for: Mu opioid receptors on vGluT2‐expressing glutamatergic neurons modulate opioid reward
Source: Addict Biol. 2020 Jul 20;26(3):e12942. doi: 10.1111/adb.12942 (PMC7854952; doi:10.1111/adb.12942)
Supplement: Supplementary file 1 — Figure S1. MORflox‐vGluT2cre mice acquire ethanol CPP. Ethanol (EtOH) CPP was performed as previously described for oxycodone CPP, except there were 4 conditioning sessions for saline and ethanol (3 g/kg i.p.). A) Following conditioning with 3 g/kg ethanol, Cre− (Ctrl) and Cre+ (KO) mice spent more time in the EtOH‐paired side (two‐way ANOVA: Genotype, p=0.86, F (1, 28) = 0.033, Session: p=0.0002, F (1, 28) = 19.07, Interaction, p=0.28, F (1, 28) = 1.24) (Sidak's post hoc tests. Session: Cre+ (KO) p=0.031, Cre− (Ctrl), p=0.0029). n=18 Cre+ (KO), 12 Cre− (Ctrl). B) Cre− (Ctrl) and Cre+ (KO) mice showed conditioned place preference for 3 g/kg EtOH. (Unpaired t‐test: p=0.76, t=0.30, df=28). Data collapsed across sex. ***p<0.001. Error bars indicate +/‐ SEM. Cre+ (KO)= MORflox‐vGluT2cre positive (+); Cre– (Ctrl)= MORflox‐vGluT2cre negative (‐). S2. C57BL/6J mice orally consume oxycodone. A) Adult male C57Bl/6J mice orally consumed oxycodone (1 mg/mL dissolved in reverse osmosis water) over twelve 24 hr sessions (n=8). B) C57BL/6J mice equally preferred oxycodone and water (one‐sample t‐test, theoretical mean=50, p=0.84, t=0.21, df=6). Error bars indicate +/‐ SEM. Figure S3 Associated with Figure 4. A) There were no differences between genotypes in total fluid consumption during oxycodone drinking sessions (two‐way ANOVA: Genotype, p=0.16, F (1, 31) = 2.088, Concentration, p=0.032, F (1.582, 49.03) = 4.052, Interaction, p=0.17, F (3, 93) = 1.732). n=16 (8M/8F) Cre+ (KO); n=17 (9M/8F) Cre− (Ctrl). B) There were no genotype differences in sucrose consumption, although there was a significant genotype x sucrose concentration interaction (two‐way ANOVA: Genotype, p=0.22, F (1, 31) = 1.55; Concentration, p<0.0001, F (1.751, 54.27) = 69.14; Concentration x Genotype, p=0.0083, F (2, 62) = 5.18) (Sidak's post hoc tests. Genotype: 0.5% p=0.25; 1% p=0.071; 2% p=0.99). n=16(9M/7F) Cre+ (KO), 17(8M/9F) Cre− (Ctrl). C) There were no differences between genotypes in fluid consumption [file ADB-26-e12942-s001.docx]

**SUPPLEMENTAL INFORMATION**

**Mu opioid receptors on vGluT2-expressing glutamatergic neurons modulate opioid reward**

Kaitlin C. Reeves^1^, Megan J. Kube^1^, Gregory G. Grecco^1,2^, Brandon M. Fritz^1^, Braulio Muñoz^1^, Fuqin Yin^1^, Yong Gao^1^, David L. Haggerty^1^, Hunter J. Hoffman^1^, Brady K. Atwood^1,3,*,^

^1^ Department of Pharmacology and Toxicology, Indiana University School of Medicine, Indianapolis, IN, 46202, USA.

^2^ Indiana University School of Medicine, Medical Scientist Training Program, Indianapolis, IN 46202

^3^ Stark Neurosciences Research Institute, Indiana University School of Medicine, Indianapolis, IN, 46202, USA.

*Correspondence: 320 W. 15^th^ St, NB-400C, Indianapolis, IN 46202; bkatwood@iu.edu; Phone: (317) 274-8917, Fax: (317) 274-7714.

**Supplemental Materials and Methods**

Animals

Experiments were conducted on cre-recombinase expressing mice [MORflox-vGluT2cre (+), Cre+ (KO)] and littermate controls [MORflox-vGluT2cre (-), Cre- (Ctrl)]. All mutant mice used in these studies have been backcrossed to C57BL/6J mice for a minimum of 7 generations. Mice were group-housed on a standard 12-hour light/dark cycle (lights on 0700hr), with the exception of mice used for 2BC and food consumption studies, which were single-housed on a reverse 12-hour light/dark cycle (lights on 1800 hr), with at least one week acclimation before testing. Food and water were available ad libitum for all mice. Male and female mice were between 8-12 weeks of age at the start of experiments and tested in only one experiment, except where otherwise indicated.

Brain Slice Preparation for Electrophysiology

Animals were deeply anesthetized with isoflurane before decapitation. The brain was quickly removed and placed into ice-cold cutting solution (194mM sucrose, 30mM NaCl, 4.5mM KCl, 1mM MgCl_2_, 26mM NaHCO_3_, 1.2mM NaH_2_PO_4_, 10mM Glucose) saturated with a mixture of 95% O_2_/5% CO_2_, and sliced to a thickness of 280 μm on a VT1200S vibratome (Leica, Germany). Slices containing the dorsal striatum were transferred to an artificial cerebrospinal fluid (aCSF) solution containing (124mM NaCl, 4.5mM KCl, 1mM MgCl_2_, 26mM NaHCO_3_, 1.2mM NaH_2_PO_4_, 10mM Glucose, 2mM CaCl_2_; adjusted to 310–320 mOsm, continuously bubbled with 95% O_2_/5% CO_2_,) at 32 °C for 1 hour before being moved to room temperature. Immediately before recording, slices were transferred to a recording chamber continuously perfused with aCSF solution that was saturated with 95% O_2_/5% CO2 and held at 30 °C.

Behavior Experiments

Mice involved in behavior experiments were habituated to handling for at least three days prior to testing. Mice were acclimated to the behavior room with a white noise machine for at least 30 minutes before testing.

*Open-field locomotor activity*: Mice were placed in the center of unlit open-field chambers (16” X 16”; Omnitech, Columbus, OH) and recorded for 20 minutes. Mice were acclimated to the chamber on day 1. On day 2 mice were habituated to injections by giving them a saline injection (10 mL/kg i.p.) before being placed in the chamber. Saline testing occurred on day 3; mice were given a saline injection (i.p.) immediately prior to being placed in the chamber. On day 4, mice were injected with oxycodone (5 mg/kg i.p.) before being immediately placed in the chamber. Locomotor activity was measured by recording the number of beam breaks in horizontal directions by Fusion software.

*Oxycodone conditioned place preference (CPP)*: Mice underwent pretesting in a 3-chambered apparatus (Omnitech), consisting of two contextually different chambers with different wall patterns and floor textures, connected by a center, neutral chamber. Mice were injected with saline (10 mL/kg i.p.) before being placed into the center chamber and allowed to freely explore the entire apparatus for 20 minutes. Duration of time spent on each side of the apparatus was measured using Fusion software (Omnitech). Initial side preference was determined by comparing the amount of time spent on each side of the apparatus during the pretest. For conditioning sessions, a biased design was used, where any mouse that showed an initial side preference greater than 200 seconds was given oxycodone in its initially non-preferred side. The side of the drug-paired chamber was counterbalanced across genotypes. Conditioning sessions, where mice were confined to one of the contextually distinct chambers, occurred twice a day for three days. In the mornings, mice were injected with saline (10 mL/kg i.p.) and immediately placed into one side of the apparatus for 5 minutes. 4 hours later, mice were injected with oxycodone (0.5 or 5 mg/kg i.p.) and placed into the other side of the apparatus for 5 minutes. The day after the last conditioning session, mice were tested for conditioned place preference, using the same protocol as the pretest. Oxycodone-paired side preference was determined by comparing the time spent in the oxycodone-paired side during testing to baseline, as well as subtracting the duration of time spent in the saline-paired side from the duration of time spent in the oxycodone-paired side.

*Naloxone-precipitated withdrawal:* Mice used to test naloxone-precipitated withdrawal had previously undergone CPP with 0.5 mg/kg oxycodone. Three days following the completion of CPP testing, oxycodone was administered using a dose ramping schedule. Mice were subcutaneously injected twice daily with 10 mg/kg oxycodone on day 1, 20 mg/kg oxycodone on day 2, and 30 mg/kg oxycodone on days 3-7. All doses were administered subcutaneously during the light cycle, approximately 7 hours apart. Mice were habituated to empty cages used for withdrawal testing on the morning of day 7 for 10 minutes. On day 8, the day of testing, a final dose of oxycodone (40 mg/kg, s.c.) was administered to all mice. This slightly higher dose was chosen to ensure sufficient oxycodone levels were maintained for the remainder of behavioral testing. Approximately 60 minutes following this final oxycodone injection, mice were given a saline injection (10 mL/kg, i.p.) and placed into a clean home cage with bedding removed to observe opioid withdrawal behaviors. Following the ten-minute observation period, mice were returned to their home cage for approximately 90 minutes. Mice were then treated with the MOR antagonist, naloxone (5 mg/kg, i.p.), to precipitate opioid withdrawal and observed for another ten minutes in the same modified home cage. Opioid withdrawal-related behaviors were assessed by the same trained, blinded observer. The number of paw shakes, wet dog shakes, and jumps were counted. Ptosis, body tremor, teeth chattering, piloerection, and diarrhea were assessed on a yes/no basis with a point given for each five-minute interval that the behavior occurred during. A global withdrawal score was calculated to give all withdrawal behaviors proportional weighting using the equation: (global withdrawal score=jumps*0.8+wet dogs shakes*1+diarrhea*1.5+paw shakes*0.35+ptosis*1.5+teeth chattering*1.5+body tremor*1.5+piloerection*1.5).

*Nociception/shock-flinch:* Mice first received a saline injection (10 mL/kg s.c.). 30 min later, they were placed into startle response chambers (San Diego Instruments, San Diego, CA) that contained shock grids and received a 3 min habituation period in which 3, 115 dB (40 ms) acoustic startle pulses were given at the end of each 60 sec period. The purpose of these 115 dB pulses is to familiarize mice to the loud startle pulses and to minimize large startle responses to low intensity startle pulses. Following this, mice were exposed to a startle/shock threshold session which consisted of 5 blocks of different auditory or sensory (shock) stimuli with the auditory stimuli ranging from 85-115 dB in 10 dB increments and the shock intensities (0.08, 0.1, 0.2, 0.4, and 0.6 mA). Each block contained each auditory and shock intensity in a randomized order. The auditory stimuli lasted 40 ms and the shocks lasted 0.5 sec. The intertrial intervals were 30 sec. In total, each block contained 9 stimulus presentations. An accelerometer detects changes in force due to jumping/flinching and the output is an excitation voltage change in millivolts (mV). One week later this test was repeated after mice received an oxycodone injection (3 mg/kg s.c.).

*Sucrose/quinine two-bottle choice (2BC)*: Mice were habituated to drinking from self-made ball-bearing sipper tubes filled with reverse-osmosis water for 23 hours. Mice were given access to two sipper tubes for 23 hours, beginning 11 hours into the dark cycle: one filled with reverse-osmosis water and the other filled with either sucrose or quinine solution. Mice were counterbalanced across genotypes into groups receiving sucrose or quinine first.  Following 6 testing sessions, a 1-week washout period with no drinking sessions was conducted before switching substances. Increasing concentrations of sucrose (0.5%, 1%, 2% w/v) or quinine (0.03 mM, 0.1 mM, 0.3 mM) were given after two sessions at the previous concentration. Sipper tubes were presented on alternating sides of the home cage to account for side-preference. Preference for sucrose or quinine over water was calculated as the percentage of total fluid consumed that contained sucrose or quinine solutions.

*Oxycodone 2BC*: Mice were habituated to drinking from sipper tubes as described above. Every other day following habituation, mice were presented with both a sipper tube filled with oxycodone solution and a sipper tube filled with reverse-osmosis water for 24 hours at a time, in their home cages, beginning 11 hours into the dark cycle. This was repeated every other day. A pilot experiment was first run with a single concentration of oxycodone (1 mg/mL) with C57Bl/6J mice **(Fig. S2)**. Experiments with transgenic mice were performed with increasing concentrations of oxycodone given after 4 sessions at the previous concentration (0.1 mg/mL, 0.3 mg/mL, 1 mg/mL, 3 mg/mL). Sipper tubes were presented on alternating sides of the home cage to account for side-preference. Preference for oxycodone over water was calculated as the percentage of total fluid consumed that contained oxycodone solution.

**Supplemental figures**

**
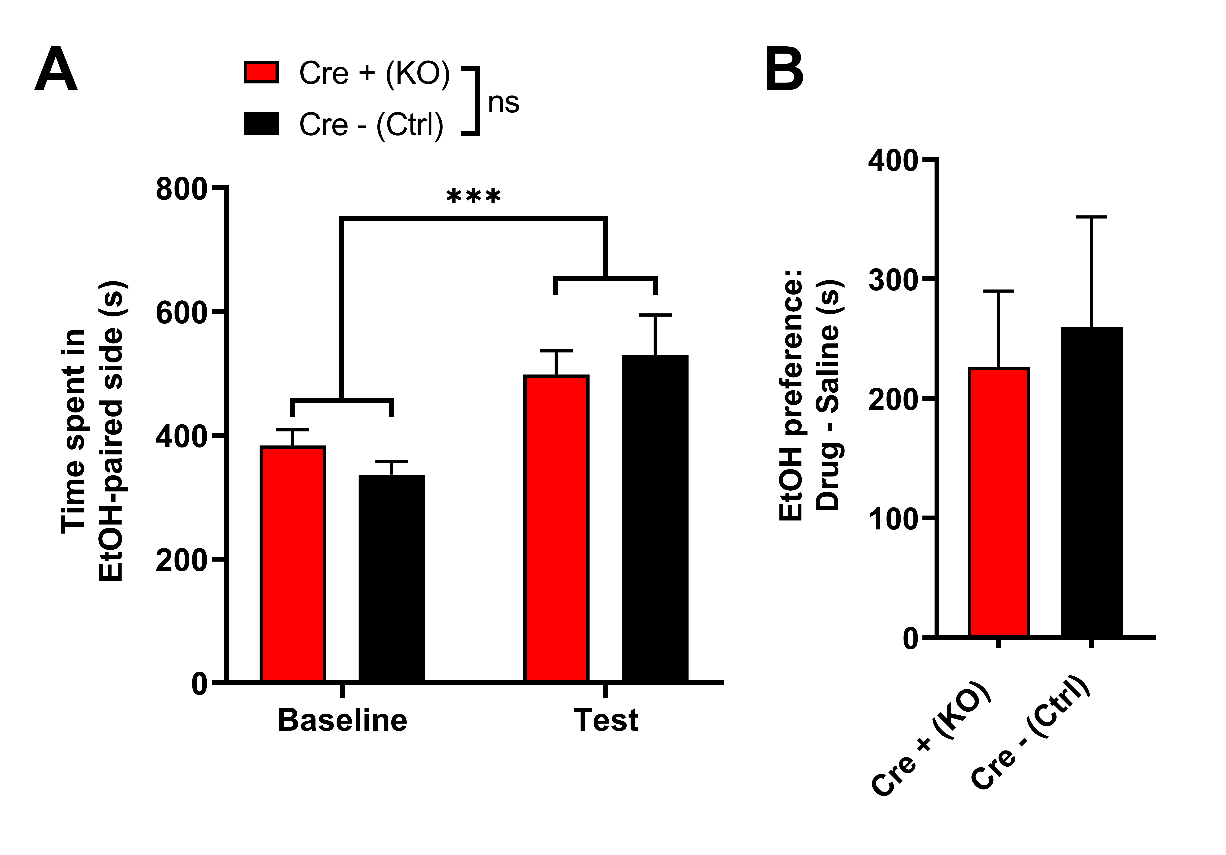
**

**Supplemental Figure S1. MORflox-vGluT2cre mice acquire ethanol CPP.** Ethanol (EtOH) CPP was performed as previously described for oxycodone CPP, except there were 4 conditioning sessions for saline and ethanol (3 g/kg i.p.). **A)** Following conditioning with 3 g/kg ethanol, Cre- (Ctrl) and Cre+ (KO) mice spent more time in the EtOH-paired side (two-way ANOVA: Genotype, p=0.86, F (1, 28) = 0.033, Session: p=0.0002, F (1, 28) = 19.07, Interaction, p=0.28, F (1, 28) = 1.24) (Sidak’s post hoc tests. Session: Cre+ (KO) p=0.031, Cre- (Ctrl), p=0.0029). n=18 Cre+ (KO), 12 Cre- (Ctrl). **B)** Cre- (Ctrl) and Cre+ (KO) mice showed conditioned place preference for 3 g/kg EtOH. (Unpaired t-test: p=0.76, t=0.30, df=28). Data collapsed across sex. ***p<0.001. Error bars indicate +/- SEM. Cre+ (KO)= MORflox-vGluT2cre positive (+); Cre– (Ctrl)= MORflox-vGluT2cre negative (-).

**
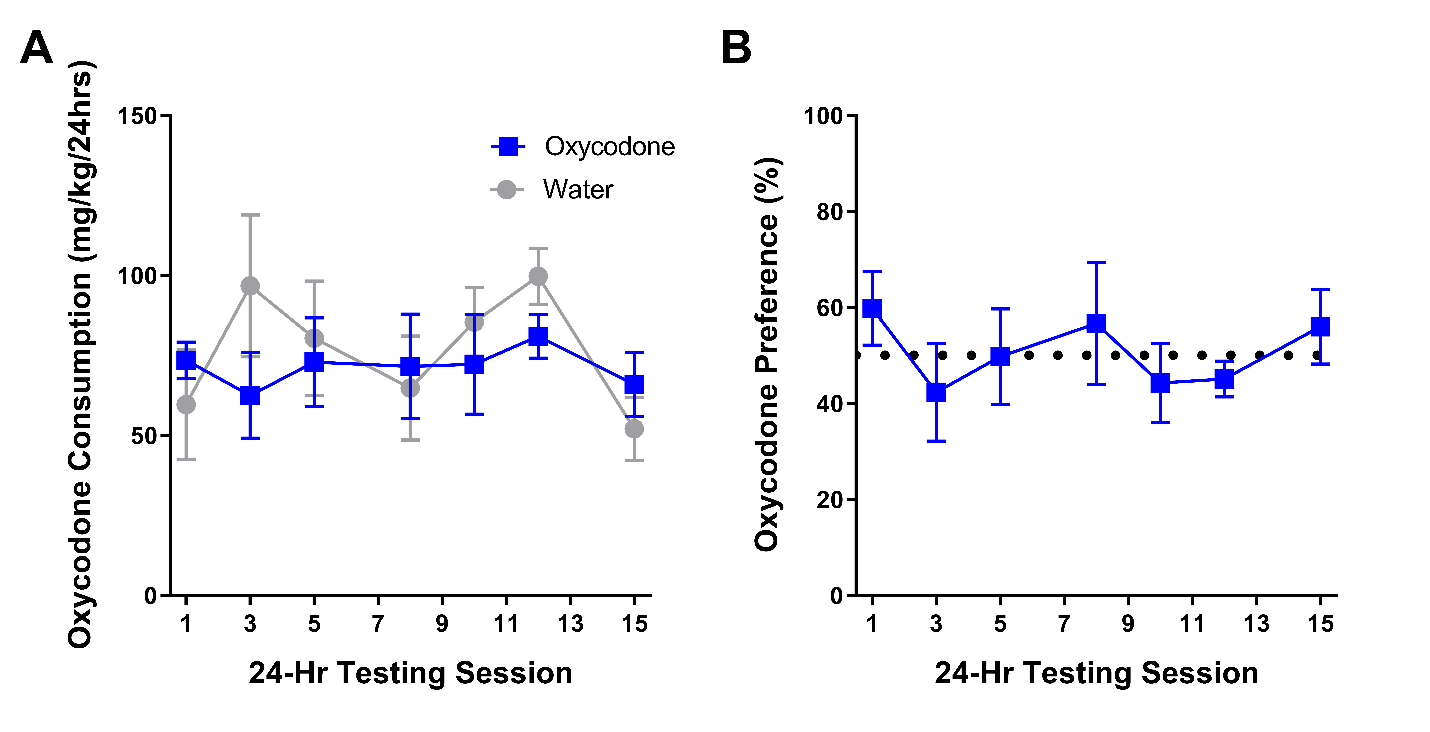
**

**Supplemental Figure S2. C57BL/6J mice orally consume oxycodone.** **A)** Adult male C57Bl/6J mice orally consumed oxycodone (1 mg/mL dissolved in reverse osmosis water) over twelve 24 hr sessions (n=8). **B)** C57BL/6J mice equally preferred oxycodone and water (one-sample t-test, theoretical mean=50, p=0.84, t=0.21, df=6). Error bars indicate +/- SEM.


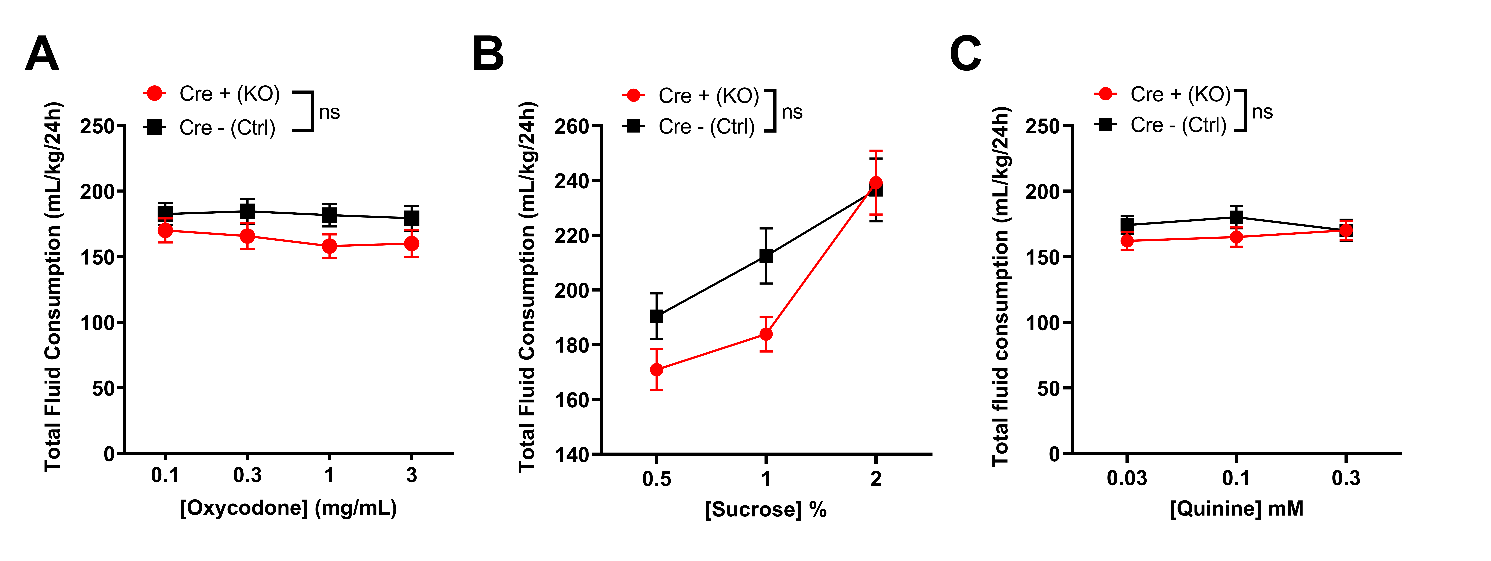


**Supplemental Figure S3** Associated with Figure 4. **A)** There were no differences between genotypes in total fluid consumption during oxycodone drinking sessions (two-way ANOVA: Genotype, p=0.16, F (1, 31) = 2.088, Concentration, p=0.032, F (1.582, 49.03) = 4.052, Interaction, p=0.17, F (3, 93) = 1.732). n=16 (8M/8F) Cre+ (KO); n=17 (9M/8F) Cre- (Ctrl). **B)** There were no genotype differences in sucrose consumption, although there was a significant genotype x sucrose concentration interaction (two-way ANOVA: Genotype, p=0.22, F (1, 31) = 1.55; Concentration, p<0.0001, F (1.751, 54.27) = 69.14; Concentration x Genotype, p=0.0083, F (2, 62) = 5.18) (Sidak’s post hoc tests. Genotype: 0.5% p=0.25; 1% p=0.071; 2% p=0.99). n=16(9M/7F) Cre+ (KO), 17(8M/9F) Cre- (Ctrl). **C)** There were no differences between genotypes in fluid consumption during quinine drinking sessions (two-way ANOVA: Genotype, p=0.39, F (1, 31) = 0.78, Concentration, p=0.32, F (1.93, 59.84) = 1.14, Concentration x Genotype, p=0.030, F (2, 62) = 3.72). n=16(9M/7F) Cre+ (KO), 17(8M/9F) Cre- (Ctrl). Data collapsed across sex. Error bars indicate +/- SEM. Cre+ (KO)= MORflox-vGluT2cre positive (+); Cre– (Ctrl)= MORflox-vGluT2cre negative (-).

**
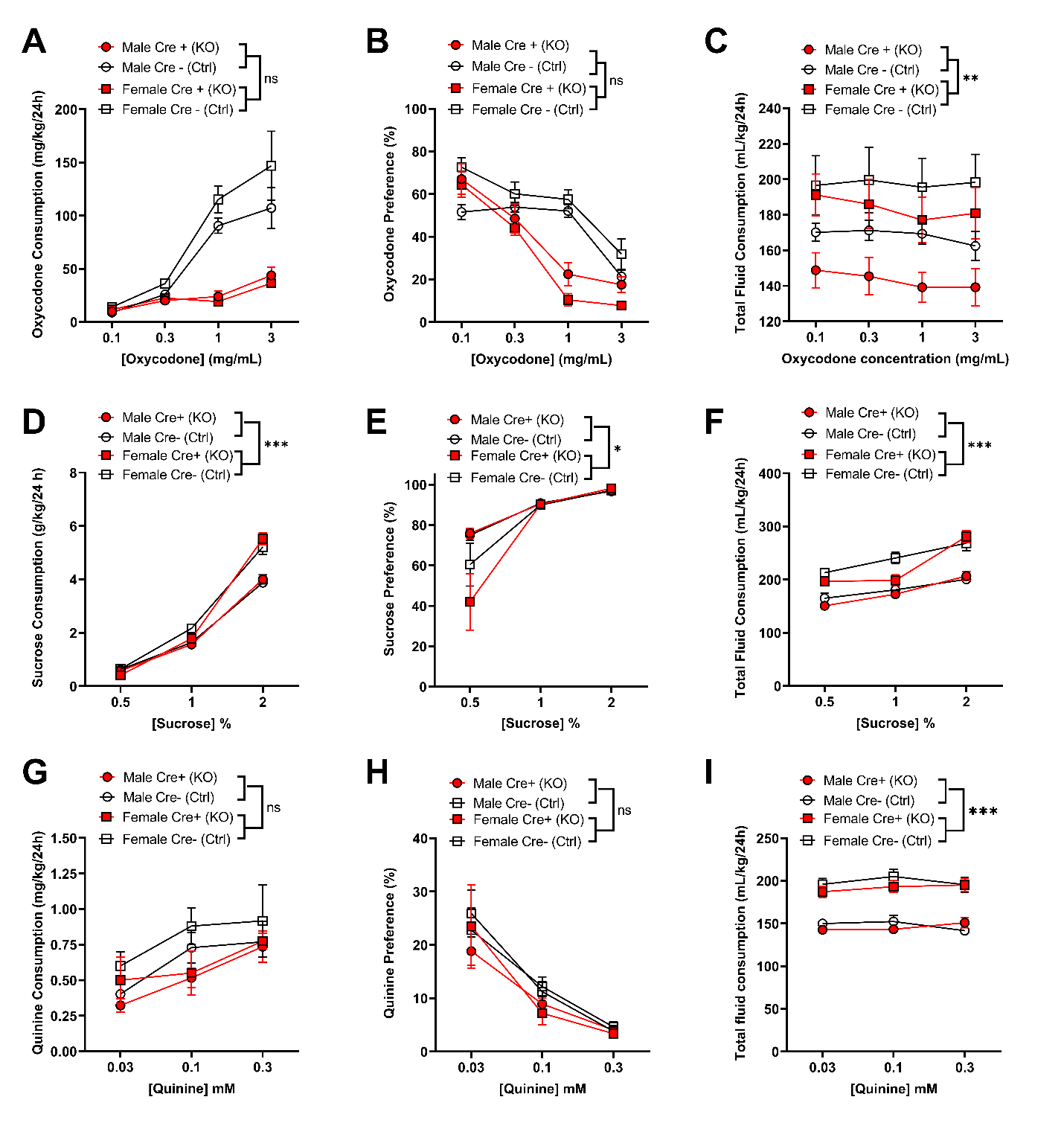
**

**Supplemental Figure S4.** Associated with Figure 4 and Supplemental Table 1. **A-C)** Assessments of sex and genotype differences in oral oxycodone consumption. n=16 (8M/8F) Cre+ (KO); n=17 (9M/8F) Cre- (Ctrl). Females consumed significantly more fluid than males. **D-F)** Assessments of sex and genotype differences in oral sucrose consumption. n=16(9M/7F) Cre+ (KO), 17(8M/9F) Cre- (Ctrl). Females consumed significantly more sucrose and total fluid than males but had lower preference for sucrose than males. **G-I)** Assessments of sex and genotype differences in oral quinine consumption. n=16(9M/7F) Cre+ (KO), 17(8M/9F) Cre- (Ctrl). Females consumed significantly more fluid than males. *p<0.05, **p<0.01, ***p<0.001. Error bars indicate +/- SEM. Cre+ (KO)= MORflox-vGluT2cre positive (+); Cre– (Ctrl)= MORflox-vGluT2cre negative (-).

**
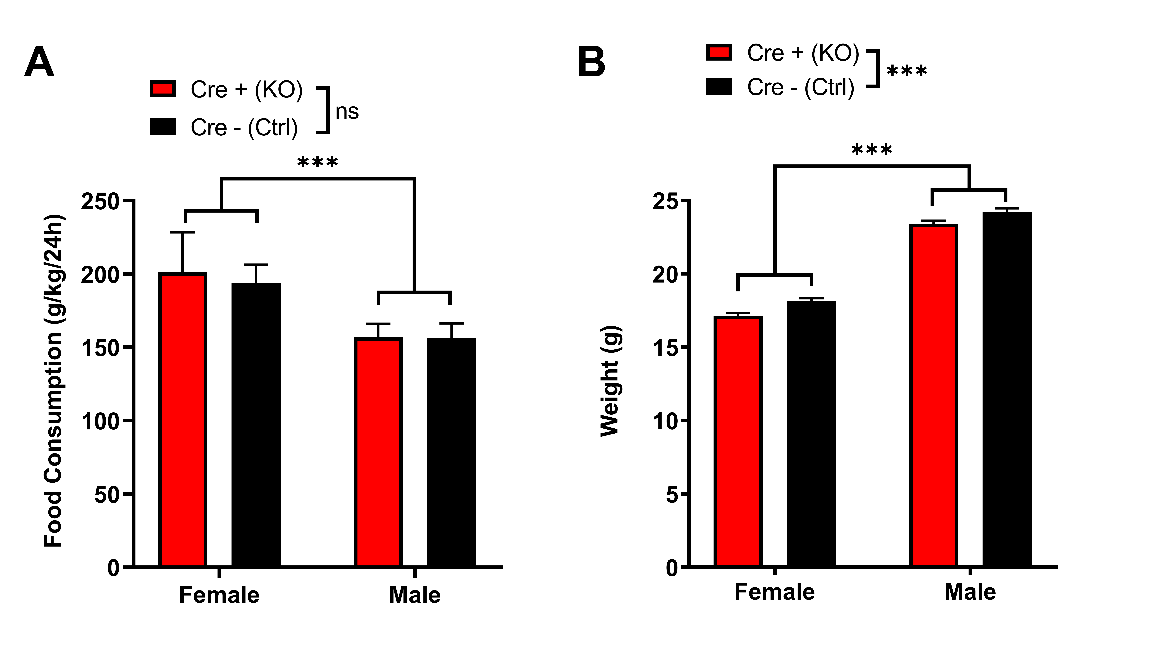
**

**Supplemental Figure S5**

**A)** Food consumption between Cre+ (KO) and Cre- (Ctrl) mice. Female mice of both genotypes consumed more food than males (two-way ANOVA: Genotype, p=0.46, F (1, 33) = 0.57; Sex, p<0.0001, F (1, 33) = 60.46; Interaction, p=0.56, F (1, 33) = 0.35) (Sidak’s post hoc tests. Genotype: Female p=0.56, Male p=0.99; Sex: Cre+ (KO) p<0.0001, Cre- (Ctrl) p<0.0001). n=16 (8M/8F) Cre +(KO), 21 (9M/12F) Cre- (Ctrl) mice. **B)** Cre+ (KO) mice weighed less at baseline (~8 wks old) than Cre- (Ctrl) mice. Females weighed less than males in both genotypes (two-way ANOVA: Genotype, p<0.0001, F (1, 277) = 21.21; Sex, p<0.0001, F (1, 277) = 938.9; Interaction, p=0.58, F (1, 277) = 0.32) (Sidak’s post hoc tests. Genotype: Female p=0.0095, Male p=0.0006; Sex: Cre+ (KO) p<0.0001, Cre- (Ctrl) p<0.0001). n=139 (70M/69F) Cre+ (KO), 142 (69M/73F) Cre- (Ctrl). ***p<0.001. Error bars indicate +/- SEM. Cre+ (KO)= MORflox-vGluT2cre positive (+); Cre– (Ctrl)= MORflox-vGluT2cre negative (-).


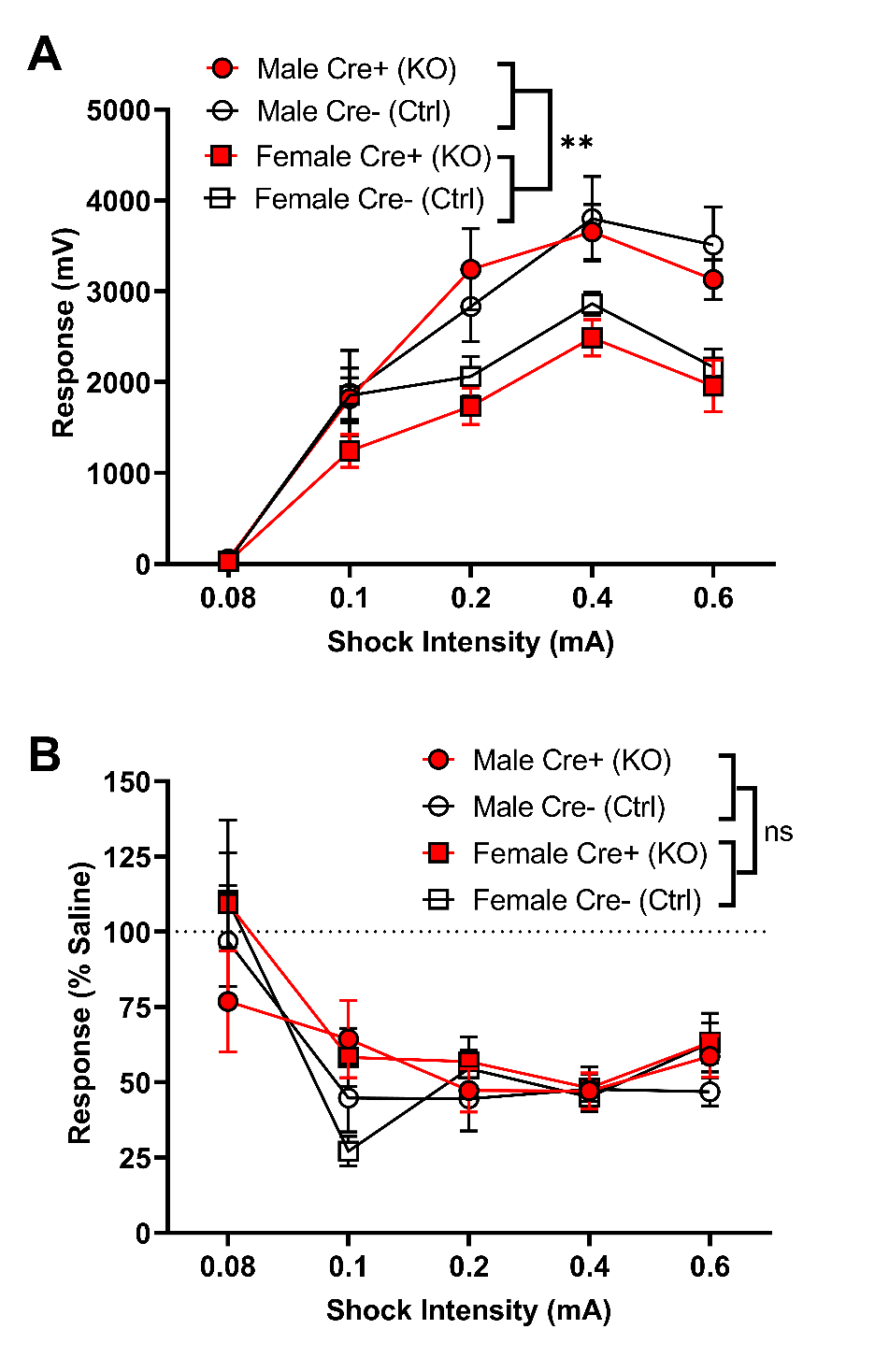


**Supplemental Figure S6. Male mice are more sensitive to shock stimuli than female mice.** Associated with Figure 5. n=17 (9M/8F) Cre+ (KO), 18(8M/10F) Cre- (Ctrl) for all panels. **A)** There are no genotype differences in baseline responses to shock stimuli, but male mice are more sensitive to shock stimuli than female mice (Residual maximum likelihood analysis: Shock intensity, p<0.0001, F(4,36)=83.9, Sex, p=0.0019, F(1,9)=18.91, Genotype, p=0.36, F(1,9)=0.92, Shock intensity x Sex, p=0.0034, F(4,36)=4.77, Shock intensity x Genotype, p=0.79, F(4,36)=0.42, Sex x Genotype, p=0.57, F(1,9)=0.36, Shock intensity x Sex x Genotype, p=0.45, F(4,10)=1.0). **B)** Behavioral responses to oxycodone treatment were not impacted by the sex or genotype of the mice. There was a significant effect of shock intensity. (Residual maximum likelihood analysis: Shock Intensity, p<0.0001, F (2.095, 65.46) = 16.19, Sex, p=0.2863, F (1, 32) = 1.176, Genotype, p=0.3947, F (1, 32) = 0.7444, Shock Intensity x Sex, p=0.1994, F (4, 125) = 1.523, Shock Intensity x Genotype, p=0.1981, F (4, 125) = 1.528, Sex x Genotype, p=0.7041, F (1, 32) = 0.1468, Shock Intensity x Sex x Genotype, p=0.8821, F (4, 125) = 0.2930). **p<0.01. Error bars indicate +/- SEM. Cre+ (KO)= MORflox-vGluT2cre positive (+); Cre– (Ctrl)= MORflox-vGluT2cre negative (-).

**
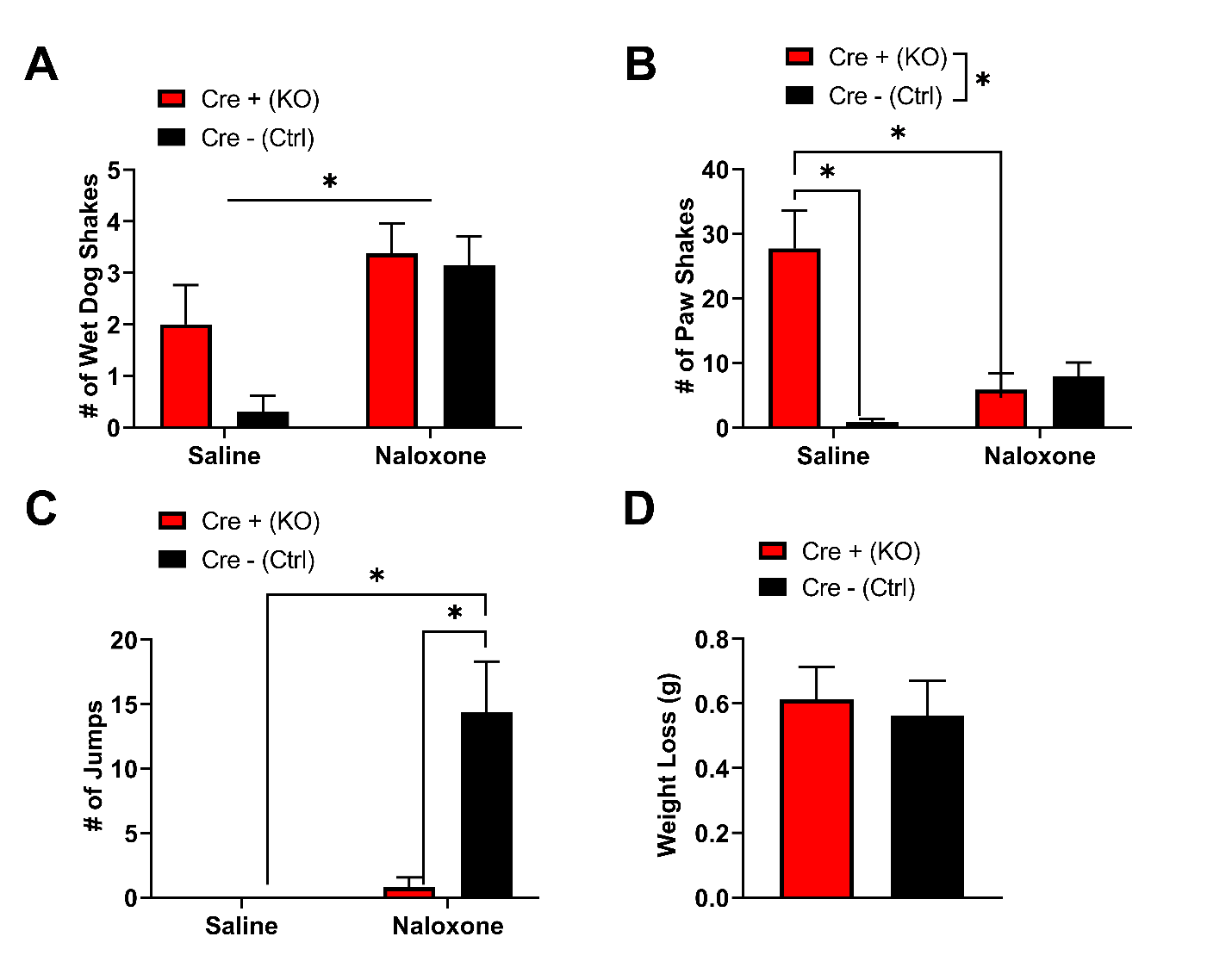
Supplemental Figure S7. Individual oxycodone withdrawal-related behavioral assessments.** Associated with Figure 6. n=16(8M/8F) Cre+ (KO), 13(5M/8F) Cre- (Ctrl) for all panels. **A)** Naloxone treatment produced an increase in wet dog shakes in oxycodone-dependent Cre- (Ctrl) mice, but not in Cre+ (KO) mice. (two-way ANOVA: Genotype, p=0.1057, F (1, 27) = 2.803; Treatment, p=0.0028, F (1, 27) = 10.80; Treatment x Genotype, p=0.2621, F (1, 27) = 1.312) (Sidak’s post-hoc, Treatment: Cre+ (KO), p=0.23; Cre- (Ctrl), p=0.012). **B)** Oxycodone-dependent Cre+ (KO) mice displayed more paw shakes than Cre- (Ctrl) following saline treatment, but naloxone treatment reduced these. (two-way ANOVA: Genotype, p=0.0034, F (1, 27) = 10.33; Treatment, p=0.0457F (1, 27) = 4.390; Treatment x Genotype, p=0.0004, F (1, 27) = 16.54) (Sidak’s post hoc tests. Genotype: Saline p<0.0001, Naloxone p=0.91; Treatment: Cre+ (KO) p=0.0002, Cre- (Ctrl) p=0.35). **C)** Naloxone treatment increased jumping behavior in oxycodone-dependent Cre- (Ctrl) mice, but not in Cre+ (KO) mice. (two-way ANOVA: Genotype, p=0.0009, F (1, 27) = 14.05; Treatment, p=0.0003, F (1, 27) = 17.61; Treatment x Genotype, p=0.0009, F (1, 27) = 14.05) (Sidak’s post-hoc tests. Genotype: Saline p>0.9999, Naloxone p<0.0001; Treatment, Cre + (KO) p=0.93, Cre– (Ctrl) p<0.0001). **D)** Oxycodone-dependency did not result in differences in weight loss between genotypes. (unpaired t-test, p=0.74, t=0.3425, df=27). No sex differences were detected. Data are collapsed across sex. *p<0.05. Error bars indicate +/- SEM. Cre+ (KO)= MORflox-vGluT2cre positive (+); Cre– (Ctrl)= MORflox-vGluT2cre negative (-).

**Supplemental Tables**

|  | **Concentration** | **Sex** | **Genotype** | **Concentration x Sex** | **Concentration x Genotype** | **Sex x Genotype** | **Concentration x Sex x Genotype** |
| --- | --- | --- | --- | --- | --- | --- | --- |
| **Oxycodone** | | | | | | | |
| mg/kg | F (1.28, 37.18) = 47.70 | F (1, 29) = 1.85 | F (1, 29) = 45.54 | F (3, 87) = 0.33 | F (3, 87) = 22.78 | F (1, 29) = 2.75 | F (3, 87) = 1.11 |
|  | P<0.0001 | P=0.19 | P<0.0001 | P=0.81 | P<0.0001 | P=0.11 | P=0.35 |
| Pref. | F (2.58, 74.78) = 117.4 | F (1, 29) = 0.28 | F (1, 29) = 18.69 | F (3, 87) = 2.22 | F (3, 87) = 24.11 | F (1, 29) = 6.90 | F (3, 87) = 0.60 |
|  | P<0.0001 | P=0.60 | P=0.0002 | P=0.092 | P<0.0001 | P=0.014 | P=0.62 |
| Total Fluid | F (1.57, 45.46) = 3.84 | F (1, 29) = 9.13 | F (1, 29) = 2.82 | F (3, 87) = 0.63 | F (3, 87) = 1.69 | F (1, 29) = 0.24 | F (3, 87) = 0.35 |
|  | P=0.038 | P=0.0052 | P=0.10 | P=0.60 | P=0.18 | P=0.63 | P=0.79 |
| **Sucrose** | | | | | | | |
| mg/kg | F (1.40, 40.59) = 1200 | F (1, 29) = 31.99 | F (1, 29) = 0.26 | F (2, 58) = 40.40 | F (2, 58) = 3.68 | F (1, 29) = 0.25 | F (2, 58) = 1.10 |
|  | P<0.0001 | P<0.0001 | P=0.62 | P<0.0001 | P=0.031 | P=0.61 | P=0.34 |
| Pref. | F (1.048, 30.39) = 56.69 | F (1, 29) = 6.32 | F (1, 29) = 0.75 | F (2, 58) = 8.75 | F (2, 58) = 1.20 | F (1, 29) = 0.87 | F (2, 58) = 1.49 |
|  | P<0.0001 | P=0.018 | P=0.39 | P=0.0005 | P=0.31 | P=0.36 | P=0.23 |
| Total Fluid | F (1.70, 49.16) = 84.09 | F (1, 29) = 49.92 | F (1, 29) = 1.85 | F (2, 58) = 5.51 | F (2, 58) = 7.47 | F (1, 29) = 0.42 | F (2, 58) = 2.67 |
|  | P<0.0001 | P<0.0001 | P=0.18 | P=0.0064 | P=0.0013 | P=0.52 | P=0.078 |
| **Quinine** | | | | | | | |
| mg/kg | F (1.58, 45.90) = 9.028 | F (1, 29) = 1.85 | F (1, 29) = 2.68 | F (2, 58) = 0.22 | F (2, 58) = 0.83 | F (1, 29) = 0.20 | F (2, 58) = 0.055 |
|  | P=0.0012 | P=0.18 | P=0.11 | P=0.80 | P=0.44 | P=0.66 | P=0.95 |
| Pref. | F (1.15, 33.38) = 52.39 | F (1, 29) = 0.084 | F (1, 29) = 1.42 | F (2, 58) = 1.16 | F (2, 58) = 0.42 | F (1, 29) = 0.0052 | F (2, 58) = 0.048 |
|  | P<0.0001 | P=0.77 | P=0.24 | P=0.32 | P=0.66 | P=0.94 | P=0.95 |
| Total Fluid | F (1.94, 56.12) = 1.13 | F (1, 29) = 76.49 | F (1, 29) = 0.69 | F (2, 58) = 0.53 | F (2, 58) = 3.48 | F (1, 29) = 0.18 | F (2, 58) = 0.27 |
|  | P=0.33 | P<0.0001 | P=0.41 | P=0.59 | P=0.038 | P=0.67 | P=0.77 |

**Table 1. Statistical Analyses of Sex Differences in Oral Consumption of Oxycodone, Sucrose, and Quinine.** Associated with Figure 4 and Supplemental Figure 4. Assessments of consumption and preference (Pref) of each substance and total fluid intake associated with each substance. All data analyzed with three-way ANOVA with genotype, sex, and substance concentration as factors. Significant differences are marked in shaded cells.
